# Electroantennogram and machine learning reveal a volatile blend mediating avoidance behavior by *Tuta absoluta* females to a wild tomato plant

**DOI:** 10.1038/s41598-022-13125-0

**Published:** 2022-05-27

**Authors:** Raphael Njurai Miano, Pascal Mahukpe Ayelo, Richard Musau, Ahmed Hassanali, Samira A. Mohamed

**Affiliations:** 1 International Centre of Insect Physiology and Ecology (icipe), P.O Box 30772-00100, Nairobi, Kenya; 2 Department of Chemistry, Kenyatta University, P.O Box 43844-00100, Nairobi, Kenya

**Keywords:** Chemical biology, Physiology

## Abstract

Tomato cultivation is threatened by the infestation of the nocturnal invasive tomato pinworm, *Tuta absoluta*. This study was based on field observations that a wild tomato plant, *Solanum lycopersicum* var. *cerasiforme*, grown in the Mount Kenya region, Kenya, is less attacked by *T. absoluta,* unlike the cultivated tomato plants like *S. lycopersicum* (var. Rambo F1). We hypothesized that the wild tomato plant may be actively avoided by gravid *T. absoluta* females because of the emission of repellent allelochemical constituents. Therefore, we compared infestation levels by the pest in field monocrops and intercrops of the two tomato genotypes, characterized the headspace volatiles, then determined the compounds detectable by the insect through gas chromatography-linked electroantennography (GC-EAG), and finally performed bioassays using a blend of four EAG-active compounds unique to the wild tomato. We found significant reductions in infestation levels in the monocrop of the wild tomato, and intercrops of wild and cultivated tomato plants compared to the monocrop of the cultivated tomato plant. Quantitative and qualitative differences were noted between volatiles of the wild and cultivated tomato plants, and between day and night volatile collections. The most discriminating compounds between the volatile treatments varied with the variable selection or machine learning methods used. In GC-EAG recordings, 16 compounds including hexanal, (*Z*)-3-hexenol, *α*-pinene, *β*-myrcene, *α*-phellandrene, *β*-phellandrene, (*E*)-*β*-ocimene, terpinolene, limonene oxide, camphor, citronellal, methyl salicylate, (*E*)-*β*-caryophyllene, and others tentatively identified as 3,7,7-Trimethyl-1,3,5-cycloheptatriene, germacrene D and *cis*-carvenone oxide were detected by antennae of *T. absoluta* females. Among these EAG-active compounds, (*Z*)-3-hexenol, *α*-pinene, *α*-phellandrene, limonene oxide, camphor, citronellal, (*E*)-*β*-caryophyllene and *β*-phellandrene are in the top 5 discriminating compounds highlighted by the machine learning methods. A blend of (*Z*)-3-hexenol, camphor, citronellal and limonene oxide detected only in the wild tomato showed dose-dependent repellence to *T. absoluta* females in wind tunnel. This study provides some groundwork for exploiting the allelochemicals of the wild tomato in the development of novel integrated pest management approaches against *T. absoluta*.

## Introduction

The South American tomato pinworm, *Tuta absoluta* (Meyrick) (formerly known as *Phthorimaea absoluta* Meyrick) (Lepidoptera: Gelechiidae) is an economically damaging insect pest of tomato, *Solanum lycopersicum* L. (Solanaceae) in many parts of the world^1–3^. Various characteristics of *T. absoluta*, including the cryptic endophytic feeding behavior of its larvae, high reproductive rate with multiple overlapping generations, expansion in the range of host plants, strong dispersal capacity, and ability to cope with various abiotic conditions have made the insect a pest of economic importance globally^4–8^. Since its first detection outside South America in 2006 in eastern Spain^9^, *T. absoluta* has spread across many countries in Europe, Africa, Asia^1–3,6,10^, and continues to colonize new regions in America^11^. Gravid females lay eggs singly, preferentially on the apical leaves of tomato, but also on other plant parts including the medium and basal leaves, as well as on stems and fruits, and from seedling to mature stage of the plant^12,13^. Upon eclosion, the larvae penetrate plant tissues and feed voraciously on leaves, but also on the stems and fruits, causing between 80–100% yield losses to tomato crops in greenhouses and open fields when no control measures are taken^1,3,14^. Sprays of chemical insecticides with broad-spectrum active ingredients appear to be the primary control strategy in many parts of the world, although these chemicals are environmentally unsafe^1–3,14^. However, the endophytic feeding behavior of *T. absoluta* larvae and the development of resistance over time in field populations of the insect to several classes of insecticides have caused failure to control the pest (reviewed by Ref.^15^). A recent review has placed emphasis on some control alternatives (e.g., agronomic and cultural practices, biological control, biopesticide application, and use of semiochemicals) which could be used in integrated pest management (IPM) package for effective control of *T. absoluta* (reviewed by Ref.^2^).

The use of semiochemicals appears to be environmentally friendly and a key component of IPM for sustainable control of insect pests^16,17^. Semiochemicals are chemical molecules classified as pheromones or allelochemicals, respectively, when the chemicals mediate intra-specific or inter-specific interactions between the emitter organism and the receiver organism^18,19^. There has been great interest in the deployment of *T. absoluta* female sex pheromone lures to reduce the population density of conspecific males or the chance of mating by the insect^20^. However, reproduction by *T. absoluta* adults through polygyny (i.e., several females can mate with one male) and parthenogenesis (i.e., females ability to produce eggs in the absence of males) is a factor that impedes the control of the pest when using pheromones-based techniques^12,21,22^. Hence, control approaches that target *T. absoluta* females could efficiently reduce the population density of the pest, when used alone or in combination with other IPM components like pheromone-based trappings. Natural biogenic allelochemical volatiles released by plants contain chemical compounds that promote or deter interactions between females of herbivorous insects and their host plants, and thus have relevant ecological importance for pest control, specifically by targeting females of insect pests^16,23^.

Tomato cultivars influence the oviposition preference by *T. absoluta* females and the biological performance of the insect^24,25^, as well as its population density and distribution pattern in the field^26^. Proffit et al.^27^ reported that a wild tomato cultivar, *Solanum habrochaites*, was avoided by ovipositing *T. absoluta*, whereas the cultivated tomato cultivar, *Solanum lycopersicum*, like cv. santa clara was preferred by the insect. *Tuta absoluta* females were also shown to avoid landing and ovipositing on conspecific-infested tomato plants^28,29^. In recordings by gas chromatography-linked electroantennography detection (GC-EAD), antennae of *T. absoluta* females were found to detect compounds from several chemical classes including the aldehyde hexanal, the alcohol (*Ζ*)-3-hexenol, the ester methyl salicylate, and the benzenoid indole, which could be playing a role in the avoidance behavior of the insect to conspecific-infested plants^29^. The present study was built on our field surveys revealing that a wild cherry tomato, *S. lycopersicum* var. *cerasiforme,* which grows in the tea zones of the Mount Kenya region in Kenya, is not attacked by *T. absoluta,* unlike the cultivated varieties. This study aimed at determining the chemical volatiles that mediate interactions between *T. absoluta* and the wild and cultivated tomato plants. We hypothesized that *T. absoluta* females may be actively avoiding the wild tomato plant due to the emission of volatiles containing repellent constituents, unlike the cultivated tomato varieties that are attractive to the pest. This was investigated by comparing the relative infestation levels by the pest in the monocrops and intercrops of the cultivated and wild tomato plants in an open field set-up. Thereafter, headspace volatiles of the cultivated and wild tomato plants were compared by gas chromatography coupled mass spectrometry (GC–MS) and the compounds detectable by antennae of the insect were determined by GC-EAD recordings. Finally, a blend of EAG-active constituents unique to the wild tomato plant was formulated and its repellency to *T. absoluta* females was evaluated in a wind tunnel.

## Results

### Infestation levels of *Tuta absoluta* in monocrops and intercrops of the two tomato genotypes

**Figure 1:**
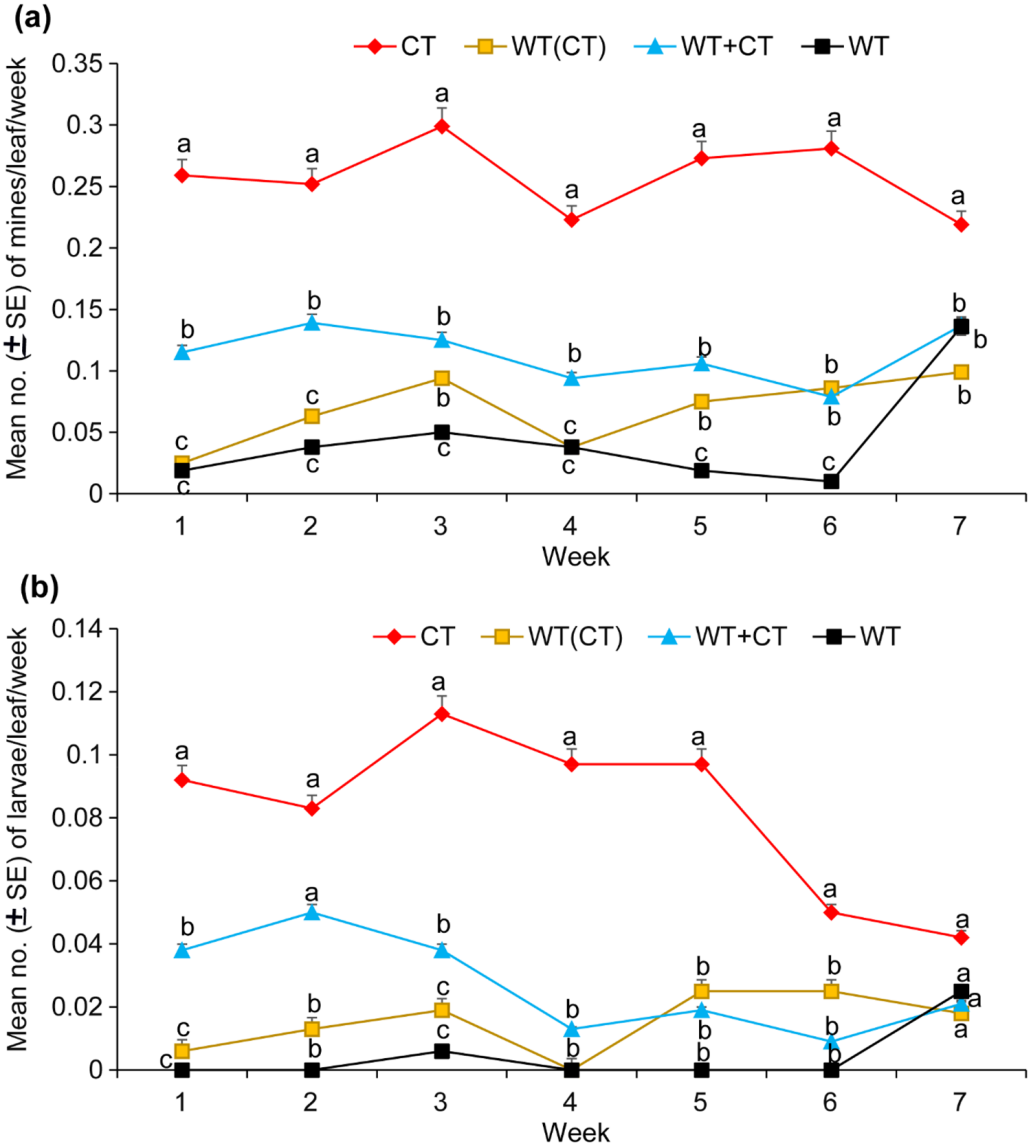
Mean numbers (± SE) of mines (**a**) and larvae (**b**) per leaf per week in the four treatments. CT = monocrop of cultivated tomato plant; WT (CT) = monocrop of the cultivated tomato plant surrounded by wild tomato plant; WT + CT = intercrop of the wild tomato and cultivated tomato plants; WT = monocrop of the wild tomato plant. For each week, bars with different letters indicate a significant difference between treatments using ANOVA and SNK tests at α = 0.05.

The results showed that the infestation levels were significantly different in all the weekly leaf samples between the monocrop of cultivated and wild tomato plants and the intercrop systems (Fig. 1). In general, the numbers of mines and larvae per leaf were not affected by the experimental block design (F_(3,9)_ = 0.544; *P* > 0.05 and F_(3,9)_ = 1.034; *P* > 0.05, respectively) but were significantly influenced by the treatment (F_(3,9)_ = 24.32; *P* < 0.001 and F_(3,9)_ = 40.06; *P* < 0.001). The mean numbers of mines and larvae in the leaves per week were highest in the monocrop of cultivated tomato (CT), intermediate in the intercrop systems [WT + CT; WT (CT)] and lowest in the monocrop of the wild tomato (WT) (Fig. 1a,b). By the seventh week, the preference for cultivated tomato plants dropped and there were no significant differences in the mean number of larvae per leaf per week between the four treatments (*P* > 0.05) (Fig. 1a). However, the mean number of mines remained higher in the monocrop of cultivated tomato (CT) than in the other treatments (Fig. 1b).

**Figure 2:**
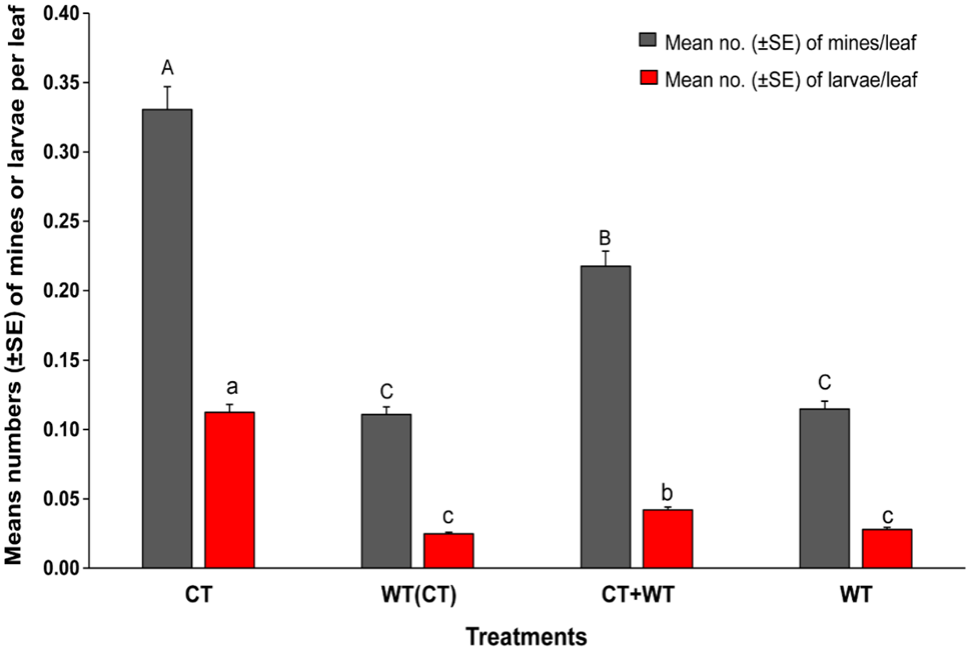
Mean numbers (± SE) of mines and larvae per leaf per treatment, CT = monocrop of the cultivated tomato plant; WT = monocrop of the wild tomato plant, WT (CT) = monocrop of the cultivated tomato plant surrounded by the wild tomato plant; and WT + CT = intercrop of the wild and cultivated tomato plants. Bars with different lowercase or uppercase letters indicate significant difference between the treatments using ANOVA and SNK tests at α = 0.05.

When the weekly samplings were pooled per treatment, the mean numbers of mines and larvae per leaf were significantly different among the cropping systems (χ^2^ = 261.2, *df* = 3, *P* < 0.001 for mines and χ^2^ = 73.04, *df* = 3, *P* < 0.001 for larvae) (Fig. 2). The numbers of mines were highest in the monocrop of cultivated tomato, followed by the intercrops of the two genotypes, then the monocrop of the cultivated tomato surrounded by the wild tomato, and lowest in the monocrop of the wild tomato (Fig. 2). Likewise, the mean numbers of larvae were higher in the monocrop of the cultivated tomato compared to the other cropping systems (Fig. 2). In the intercrops, infestation by *T. absoluta* was lower when the wild tomato was used as a border crop than when it was used as an interline crop (Fig. 2).

### Analyses of tomato headspace volatiles

The wild and cultivated tomato plants emitted distinct chemical profiles during the day and at night. A total of 74 VOCs were recorded and 61 identified, mainly comprised of monoterpenes and sesquiterpenes, and also some aldehydes, alcohols, ketones, and benzenoids (Table 1). There were both qualitative and quantitative differences in the volatiles between the two tomato genotypes, and the collection times. The wild and cultivated tomato plants emitted 74 and 69, respectively, during the day collection, while 60 and 46 were detected, respectively, in the wild and cultivated night volatile extracts. The compounds (*Z*)-3-hexenol, limonene oxide, camphor, citronellal and germacrene B were uniquely detected in headspace of the wild tomato plant. The compounds tricyclene, dill ether, *α*-terpineol, (–)-car-3-en-2-one, *α*-terpinen-7-al**,** *p*-ethyl acetophenone, *p*-cymen-7-ol, thymol**,** (*Z*)-*β*-caryophyllene**,** *α*-cedrene and four unidentified compounds (no. 8, 10, 11 and 12) were detected in the day volatiles of both wild and cultivated tomato plants but not in the night volatiles (Table 1). Quantitative differences were also found in the volatile emission between the tomato plants, and between the collection times. The total emission of volatiles was higher in the day collection than in the night collection (Table 1). The most dominant monoterpenes were *δ*-2-carene, *α*-phellandrene, *p*-cymene, and *β*-phellandrene , and the most dominant sesquiterpene was (*E*)-*β*-caryophyllene from the wild and cultivated tomato plants (Table 1). The compounds *p*-cymene, allo-ocimene, decanal, 4-(1-methylethyl)-benzaldehyde, 3-(1-methylethyl)-phenol**,** *β*-bourbonene**,** *γ*-methyl ionone, 7-methyl-3-octen-2-one, dill ether, and germacrene D were highly emitted during the day compared to the night. On the contrary, the emission rates of limonene oxide, citronellal and camphor were highest in the night volatiles of the wild tomato plant compared to the volatiles of the other treatments (Table 1). There were few quantitative variations between the wild and cultivated tomato plants. The emission rates of *α*-thujene and *δ*-3-carene were higher in the wild tomato compared to the cultivated tomato, whereas those of tricyclene and caryophyllene oxide were higher in the cultivated than in the wild tomato (Table 1).

**Table 1:** Relative mean release rates (± SE) (ng/µL/h) of volatile organic compounds detected in headspace volatiles collected during the day and at night from wild and cultivated tomato plants grown in the field (n = 4). Compounds were identified using retention times (RT), electron ionization spectrum, and retention indices calculated (RI cal) relative to relative to C_8_–C_23_ n-alkanes run on an HP-5MS, and those obtained from the literature (RI lit): (A)^30^; (B)^31^; (C)^32^; (D)^33^, as well as comparison of their spectra with the library data and published Kovats retention indices and mass spectra from online NIST library database. Compounds marked with a star (*) are those that were confirmed using available authentic standards run on an HP-5MS column. Significant *p*-values at α = 0.05 are in bold and means with different letters are significantly different based on Kruskal–Wallis ANOVA used for comparison of at least three means, while Wilcoxon paired signed-rank test was used for comparison of two means. nd = not detected.

Peak no.	RT (min)	RI cal	RI lit	Compound	Chemical category	Mean release rates (ng/µL/h) of volatile compounds				*p*-value
						Wild tomato plant		Cultivated tomato plant		
						Day	Night	Day	Night	
1	6.26	801	802^B^	Hexanal*	Aldehyde	0.06 ± 0.03	0.19 ± 0.07	0.02 ± 0.01	nd	0.146
2	7.88	863	861^D^	(*Z*)-3-Hexenol*	Alcohol	0.12 ± 0.02	0.04 ± 0.02	nd	nd	0.149
3	8.00	867	870^D^	*p*-Xylene*	Benzenoid	0.03 ± 0.01	0.03 ± 0.02	0.13 ± 0.07	nd	0.336
4	8.52	888	–	Unidentified1	–	0.39 ± 0.13	0.20 ± 0.08	0.57 ± 0.24	0.15 ± 0.05	0.337
5	8.57	890	–	Unidentified2	–	1.71 ± 0.63	1.39 ± 0.40	2.30 ± 0.86	0.79 ± 0.21	0.461
6	8.65	893	902^B^	2-Heptanol	Alcohol	0.10 ± 0.04	0.05 ± 0.02	0.04 ± 0.02	nd	0.546
7	9.48	925	927^C^	*α*-Thujene	Monoterpene	4.07 ± 1.73a	0.06 ± 0.01b	0.21 ± 0.06b	0.10 ± 0.04b	**0.028**
8	9.55	928	930^C^	Tricyclene	Monoterpene	0.17 ± 0.08b	nd	6.28 ± 1.64a	nd	**0.021**
9	9.63	931	935^C^	*α*-Pinene*	Monoterpene	23.94 ± 3.42a	9.74 ± 1.22b	21.97 ± 4.94a	19.33 ± 4.19ab	**0.045**
10	10.44	962	970^D^	3,7,7-Trimethyl-1,3,5-cycloheptatriene	Monoterpene	16.39 ± 7.78	4.14 ± 0.95	14.80 ± 3.98	6.50 ± 1.23	0.145
11	10.59	968	978^C^	*β*-Pinene*	Monoterpene	0.01 ± 0.01b	0.08 ± 0.04ab	0.12 ± 0.04a	0.21 ± 0.04a	**0.024**
12	10.66	971	980^D^	trans-Isolimonene	Monoterpene	11.73 ± 1.78a	3.12 ± 0.45b	12.67 ± 3.35a	4.68 ± 0.61ab	**0.009**
13	10.90	988	993^C^	*β*-Myrcene*	Monoterpene	2.86 ± 0.09	3.50 ± 1.25	3.83 ± 1.18	0.60 ± 0.04	0.088
14	11.03	1002	1002^D^	*δ*-2-Carene*	Monoterpene	77.55 ± 4.56	52.20 ± 5.85	61.87 ± 19.28	59.59 ± 5.64	0.134
15	11.11	1007	1005^D^	*α-*Phellandrene*	Monoterpene	35.17 ± 3.61a	11.66 ± 1.64b	34.20 ± 8.61a	21.75 ± 2.85a	**0.045**
16	11.20	1012	1011^D^	*δ*-3-Carene*	Monoterpene	14.34 ± 5.17a	1.00 ± 0.13b	2.77 ± 1.17b	0.85 ± 0.32b	**0.036**
17	11.38	1022	1018^C^	*α*-Terpinene*	Monoterpene	5.95 ± 1.89	6.12 ± 0.76	7.53 ± 3.10	9.35 ± 0.55	0.275
18	11.49	1028	1027^D^	*p*-Cymene*	Monoterpene	46.77 ± 2.22a	13.66 ± 7.48b	38.23 ± 3.22a	7.64 ± 4.78b	**0.009**
19	11.66	1032	1033^D^	*β*-Phellandrene*	Monoterpene	99.99 ± 3.74	99.32 ± 6.99	109.94 ± 5.25	96.11 ± 8.92	0.535
20	11.74	1042	1040^D^	(*Z*)-*β-*Ocimene*	Monoterpene	0.16 ± 0.02	0.73 ± 0.03	0.10 ± 0.07	0.13 ± 0.05	0.243
21	11.85	1048	–	Unidentified3	Monoterpene	1.94 ± 0.44	1.55 ± 0.33	2.23 ± 0.44	1.7 ± 0.32	0.647
22	11.91	1051	1150^D^	(*E*)-*β*-Ocimene*	Monoterpene	2.92 ± 0.79a	1.40 ± 0.38ab	2.49 ± 0.39a	0.72 ± 0.08b	**0.026**
23	12.12	1063	1061^D^	*γ*-Terpinene*	Monoterpene	3.00 ± 0.58a	1.37 ± 0.14b	0.49 ± 0.29b	2.13 ± 0.28ab	**0.049**
24	12.24	1069	–	Unidentified4	Monoterpene	1.24 ± 0.35	1.01 ± 0.24	1.46 ± 0.11	1.06 ± 0.22	0.814
25	12.38	1077	–	Unidentified5	Monoterpene	1.39 ± 0.48	1.22 ± 0.12	1.38 ± 0.33	1.45 ± 0.49	0.985
26	12.64	1091	1090^D^	Terpinolene*	Monoterpene	3.60 ± 1.29	2.17 ± 0.19	2.47 ± 0.67	3.37 ± 0.70	0.356
27	12.81	1101	1101^C^	Linalool*	Monoterpenoid alcohol	0.22 ± 0.09	0.20 ± 0.02	0.68 ± 0.21	0.46 ± 0.30	0.533
28	12.88	1105	1106^C^	Nonanal*	Aldehyde	0.36 ± 0.04a	0.42 ± 0.30a	0.38 ± 0.11a	0.02 ± 0.01b	**0.028**
29	13.02	1112	1111^B^	1,3,8-*p*-Menthatriene	Monoterpene	0.22 ± 0.04ab	0.11 ± 0.02b	0.35 ± 0.07a	0.23 ± 0.06ab	**0.029**
30	13.13	1118	–	Unidentified6	Monoterpene	0.74 ± 0.17	0.38 ± 0.04	0.61 ± 0.10	0.65 ± 0.01	0.137
31	13.30	1128	1128^D^	Allo-ocimene*	Monoterpene	0.75 ± 0.05a	0.11 ± 0.04b	0.64 ± 0.24a	0.13 ± 0.03b	**0.041**
32	13.40	1133	1138^C^	*cis-p*-Mentha-2,8-dien-1-ol	Monoterpenoid alcohol	0.56 ± 0.13	nd	0.73 ± 0.29	0.22 ± 0.15	0.232
33	13.47	1137	1137^B^	Limonene oxide*	Monoterpene	0.06 ± 0.01a	0.12 ± 0.01b	nd	nd	**0.021**
34	13.64	1146	1147^B^	Camphor*	Monoterpene	0.07 ± 0.01a	0.20 ± 0.02b	nd	nd	**0.021**
35	13.73	1151	1152^B^	Citronellal*	Monoterpenoid aldehyde	0.03 ± 0.01a	0.14 ± 0.01b	nd	nd	**0.021**
36	13.87	1159	1168^B^	4-Ethyl-benzaldehyde	Aldehyde	0.17 ± 0.01a	0.02 ± 0.01b	0.48 ± 0.29a	nd	**0.025**
37	13.89	1160	1172^C^	*p*-Mentha-1,5-dien-8-ol	Monoterpenoid alcohol	0.14 ± 0.03	0.08 ± 0.01	0.18 ± 0.06	0.19 ± 0.06	0.326
38	14.07	1170	1172^A^	(*E*)-isocitral	Aldehyde	1.08 ± 0.17a	0.20 ± 0.02b	1.09 ± 0.27a	0.26 ± 0.05b	**0.009**
39	14.23	1179	1180^D^	Dill ether	Monoterpene	2.41 ± 0.42a	0.06 ± 0.02b	3.04 ± 1.54a	0.10 ± 0.03b	**0.010**
40	14.27	1181	1188^D^	*α*-Terpineol*	Monoterpenoid alcohol	0.38 ± 0.11	nd	0.43 ± 0.28	nd	0.564
41	14.35	1185	–	Unidentified7	–	0.29 ± 0.05	nd	0.52 ± 0.25	nd	0.564
42	14.38	1187	1197^D^	Methyl salicylate*	Benzenoid ester	0.62 ± 0.06a	0.32 ± 0.14ab	0.51 ± 0.17a	0.04 ± 0.02b	**0.037**
43	14.45	1204	1206^C^	Decanal*	Aldehyde	0.40 ± 0.03a	0.05 ± 0.02b	0.41 ± 0.07a	nd	**0.024**
44	14.79	1225	1227^A^	3-(1-Methylethyl)-phenol^C^	Benzenoid alcohol	0.85 ± 0.11a	0.01 ± 0.01b	0.99 ± 0.41a	nd	**0.023**
45	15.06	1241	1241^D^	4-(1-Methylethyl)-benzaldehyde	Aldehyde	0.63 ± 0.05a	0.01 ± 0.01b	0.81 ± 0.46a	nd	**0.024**
46	15.24	1253	1254^A^	(–)-Car-3-en-2-one	Ketone	1.72 ± 0.52	nd	1.84 ± 1.19	nd	0.564
47	15.33	1258	1262^A^	*cis*-carvenone oxide	Ketone	1.16 ± 0.08	nd	1.21 ± 0.35	nd	0.387
48	15.39	1262	–	Unidentified8	Ketone	1.40 ± 0.41	nd	1.06 ± 0.71	nd	0.387
49	15.60	1275	1280^A^	*α*-Terpinen-7-al	Aldehyde	1.29 ± 0.45	nd	0.69 ± 0.19	nd	0.387
50	15.69	1281	1281^A^	*p*-Ethyl acetophenone	Ketone	0.93 ± 0.28	nd	1.19 ± 0.57	nd	0.773
51	15.80	1288	1287^A^	*p*-Cymen-7-ol	Alcohol	0.48 ± 0.08	nd	0.54 ± 0.25	nd	0.564
52	15.88	1303	1302^C^	Thymol	Monoterpenoid alcohol	0.60 ± 0.04	nd	0.71 ± 0.11	nd	0.564
53	16.03	1313	1317^A^	7-Methyl-3-octen-2-one	Ketone	1.91 ± 0.61a	0.03 ± 0.02b	2.34 ± 0.60a	nd	**0.023**
54	16.28	1331	1333^A^	5-Ethyl-2-nonen-4-one	Ketone	1.17 ± 0.14a	0.04 ± 0.01b	2.92 ± 1.43a	0.04 ± 0.03b	**0.009**
55	16.32	1334	–	Unidentified9	–	0.79 ± 0.06	nd	1.73 ± 0.64	nd	0.248
56	16.45	1343	1340^D^	*δ*-Elemene	Sesquiterpene	2.87 ± 0.85	2.36 ± 0.50	1.52 ± 0.16	1.74 ± 0.83	0.535
57	16.71	1361	1361^C^	Eugenol	Benzenoid	1.70 ± 0.29a	0.15 ± 0.05ab	1.72 ± 0.76a	0.02 ± 0.02b	**0.028**
58	16.81	1368	–	Unidentified10	Sesquiterpene	0.40 ± 0.03ab	0.12 ± 0.04b	0.84 ± 0.36a	0.03 ± 0.03c	**0.006**
59	17.00	1381	1382^C^	*α*-Copaene	Sesquiterpene	0.34 ± 0.03bc	0.10 ± 0.05 cd	0.67 ± 0.27ab	0.02 ± 0.02d	**0.010**
60	17.10	1388	1391^C^	*β*-Bourbonene	Sesquiterpene	0.25 ± 0.03a	0.04 ± 0.02b	0.40 ± 0.11a	0.02 ± 0.02b	**0.007**
61	17.19	1400	1397^C^	*β*-Elemene*	Sesquiterpene	0.78 ± 0.04a	0.33 ± 0.07b	0.87 ± 0.15a	0.40 ± 0.11b	**0.013**
62	17.43	1410	1411^C^	(*Z*)-β-Caryophyllene	Sesquiterpene	0.30 ± 0.04	nd	0.33 ± 0.06	nd	0.773
63	17.48	1422	1424^D^	*α*-Cedrene	Sesquiterpene	0.25 ± 0.05	nd	0.34 ± 0.11	nd	1
64	17.62	1431	1427^C^	(*E*)-*β*-Caryophyllene*	Sesquiterpene	14.85 ± 1.88a	9.43 ± 0.57ab	11.10 ± 1.02a	4.57 ± 1.25b	**0.009**
65	17.71	1438	1442^D^	*γ*-Elemene	Sesquiterpene	0.87 ± 0.21	0.71 ± 0.05	0.27 ± 0.16	0.51 ± 0.18	0.099
66	17.88	1450	1450^D^	6,9-Guaiadiene	Sesquiterpene	0.61 ± 0.07	0.33 ± 0.02	0.65 ± 0.14	0.34 ± 0.15	0.068
67	17.95	1456	1458^C^	Allo-aromadendrene	Sesquiterpene	0.15 ± 0.05	0.04 ± 0.03	0.29 ± 0.11	0.05 ± 0.04	0.191
68	18.05	1463	1462^D^	*α*-Humulene*	Sesquiterpene	2.66 ± 0.42a	1.94 ± 0.15a	1.79 ± 0.14ab	0.88 ± 0.28b	**0.023**
69	18.12	1468	1466^A^	*γ*-Methyl ionone	Ketone	0.90 ± 0.14a	0.14 ± 0.07b	1.15 ± 0.40a	nd	**0.024**
70	18.39	1488	1489^C^	Germacrene D	Sesquiterpene	0.48 ± 0.05a	0.12 ± 0.02b	0.77 ± 0.25a	0.12 ± 0.10b	**0.016**
71	18.66	1508	1511^C^	*β*-Bisabolene	Sesquiterpene	0.27 ± 0.02	0.15 ± 0.04	1.74 ± 0.72	0.05 ± 0.04	0.058
72	18.85	1522	1530^C^	*δ*-Cadinene	Sesquiterpene	0.09 ± 0.04	0.04 ± 0.02	0.23 ± 0.09	nd	0.329
73	19.32	1557	1559^C^	Germacrene B	Sesquiterpene	0.11 ± 0.20a	0.03 ± 0.02b	nd	nd	**0.021**
74	19.67	1595	1592^C^	Caryophyllene oxide	Sesquiterpene	0.72 ± 0.19a	0.30 ± 0.08ab	2.40 ± 1.04a	0.15 ± 0.12b	**0.035**
–	–	–	–	Total VOCs (ng/µL/h)	–	405.19 ± 31.81a	235.21 ± 19.50b	383.31 ± 35.65a	249.50 ± 18.32b	**0.009**

**Figure 3:**
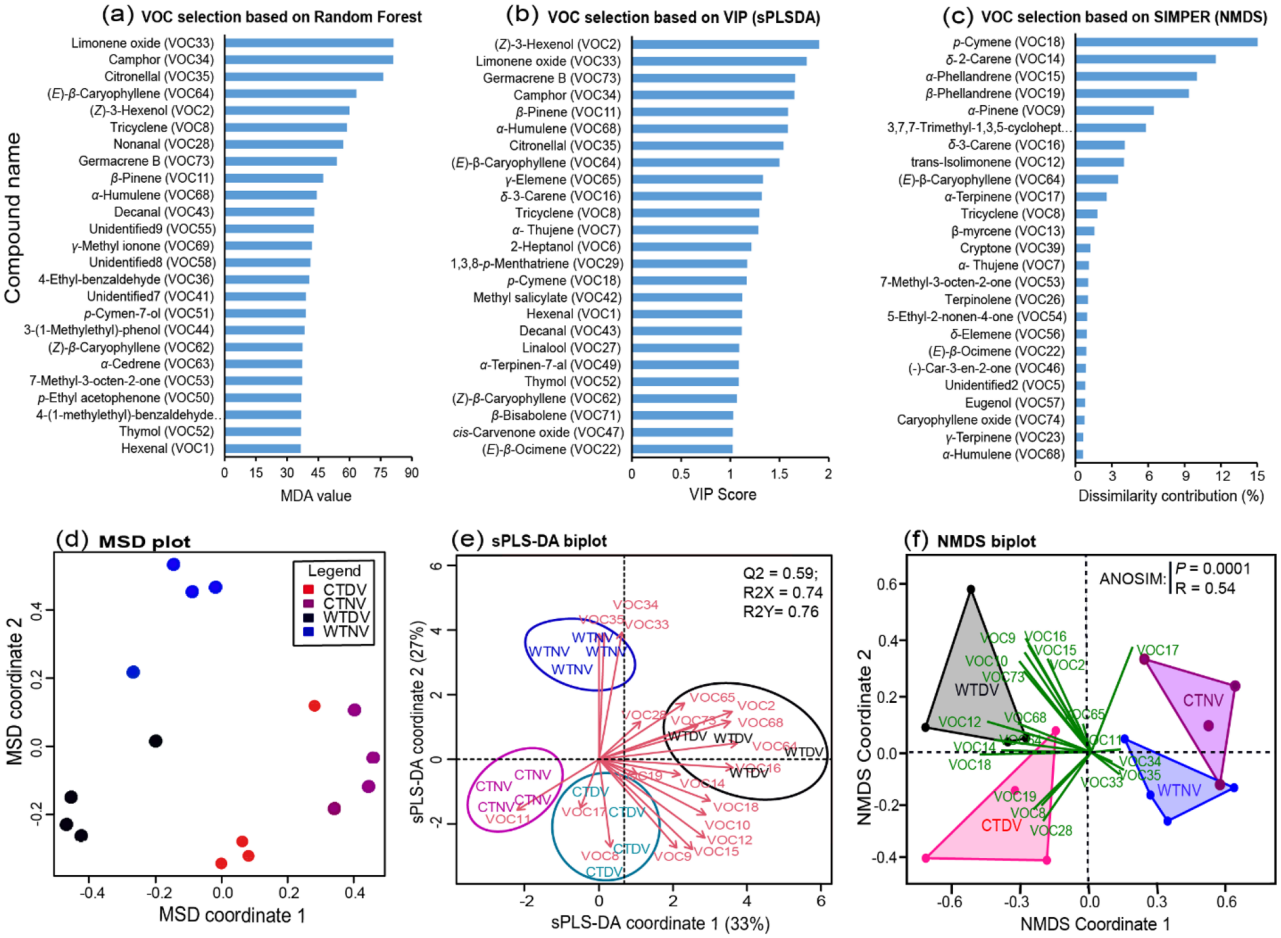
Determination of the most discriminating volatiles and their correlation with volatiles collected during the day and at night from wild and cultivated tomato plants (abbreviated as *CTDV* cultivated tomato plant day volatiles, *CTNV* cultivated tomato plant night volatiles, *WTDV* wild tomato plant day volatiles and *WTNV *wild tomato plant night volatiles). The 25 most predictive volatiles are presented in bar graphs in order of decreasing importance based on: (**a**) mean decrease in accuracy (MDA) in the random forest analysis; (**b**) variable importance in the projection (VIP > 1) from the spasce partial least square discriminant analysis (sPLS-DA); and (**c**) similarity percentage (SIMPER) of the non-metric multidimensional scaling (NMDS). Twenty discriminating VOCs were considered (i.e., a combination of the top 10 of the discriminating VOCs highlighted by the three variable selection methods (RF, sPLSDA, NMDS)). Using the release rates of the volatiles, differentiation of the volatile treatments was analyzed through: (**d**) a multidimensional scaling (MDS) ordination plot showing the distribution of the volatile treatments, (**e**) an sPLS-DA biplot and (**f**) an NMDS biplot, both showing the correlation of the discriminating VOCs with the volatile treatments.

**Figure 4:**
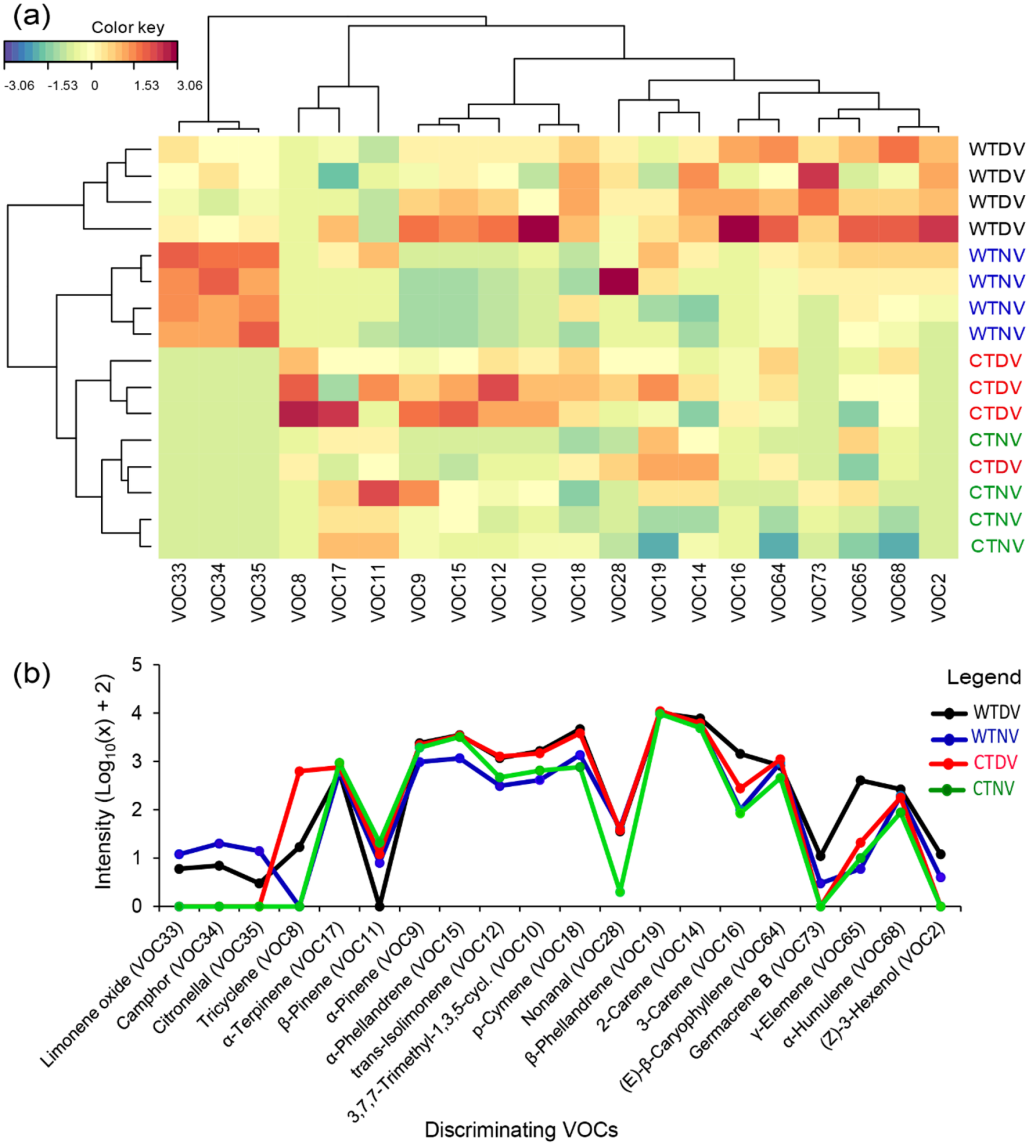
(**a**) Heatmap clustering showing the abundance (in decreasing color intensity) of the 20 discriminating VOCs across replicates of volatiles collected during the day and at night from the wild and cultivated tomato plants (abbreviated as *CTDV* cultivated tomato plant day volatiles, *CTNV* cultivated tomato plant night volatiles, *WTDV* wild tomato plant day volatiles and *WTNV* wild tomato plant night volatiles). (**b**) K-means plot showing variations in the intensity [= Log10 (x) + 2, with x = release rate of volatile)] of the 20 VOCs discriminating the volatile treatments.

The multivariate analytical tools [i.e., random forest (RF), the sparse partial least square discriminant analysis (sPLS-DA), and the non-metric multidimensional scaling (NMDS)] used for the selection of important variables showed different VOCs as discriminants between day and night volatiles of the wild and cultivated tomato plants (Fig. 3a–c). A total of 20 VOCs appeared in the top 10 of the discriminating VOCs highlighted by these methods, and were hence considered as the most discriminating between day and night volatiles of the wild and cultivated tomato plants. These discriminating VOCs included limonene oxide, citronellal, camphor, nonanal, tricyclene, germacrene B, (*E*)-*β*-caryophyllene, (*Z*)-3-hexenol, *β*-pinene, *δ*-3-carene, *γ-*elemene, *α*-terpinene, trans-isolimonene, *δ*-3-carene, 3,7,7-trimethyl-1,3,5-cycloheptatriene, α-pinene, *β*-phellandrene, *α*-phellandrene, *α*-humulene, *δ*-2-carene and *p*-cymene (Fig. 3a–c). Based on these discriminating VOCs, the multidimensional scaling (MSD), the sPLSDA and NMDS clustered the volatile treatments into four groups: (i) wild tomato plants of which volatiles were trapped during the day (WTDV), (ii) wild tomato plants of which volatiles were trapped at night (WTNV), (iii) cultivated tomato plants of which volatiles were trapped during the day (CTDV), and (iv) cultivated tomato plants of which volatiles were trapped at night (CTNV) (Fig. 3d–f). The classification accuracy of the RF analysis was very high, about 81.25%, and release rates of the most discriminating volatile compounds varied significantly across the volatile treatments (one-way ANOSIM based on Bray–Curtis dissimilarity, *p* < 0.0001, R = 0.54 for NMDS; and R2X = 0.74, R2Y = 0.76, Q2 = 0.59 for sPLS-DA). The sPLS-DA and NMDS biplots revealed that (*Z*)-3-hexenol, *δ*-3-carene, *γ*-elemene, *α*-pinene, 3,7,7-trimethyl-1,3,5-cycloheptatriene, germacrene B, and (*E*)-*β*-caryophyllene were associated with wild tomato plant day volatiles, while limonene oxide, citronellal, and camphor were associated with the wild tomato plant night volatiles. On the other hand, *β*-phellandrene, nonanal and tricyclene were associated with cultivated tomato plant day volatiles (Fig. 3e,f). The clustering heatmap and k-means plot (line graph) showed that the most discriminating VOCs are abundant in replicates of volatiles collected during the day from the wild and cultivated tomato plants than in those collected at night, except for limonene oxide, citronellal, camphor which were abundant in the night volatiles of the wild tomato plant; and *β*-pinene, *δ*-2-carene, *β*-phellandrene, and *α*-terpinene which did not show variation between day and night collections (Fig. 4a,b).

### Determination of the electrophysiologically-active compounds

**Figure 5:**
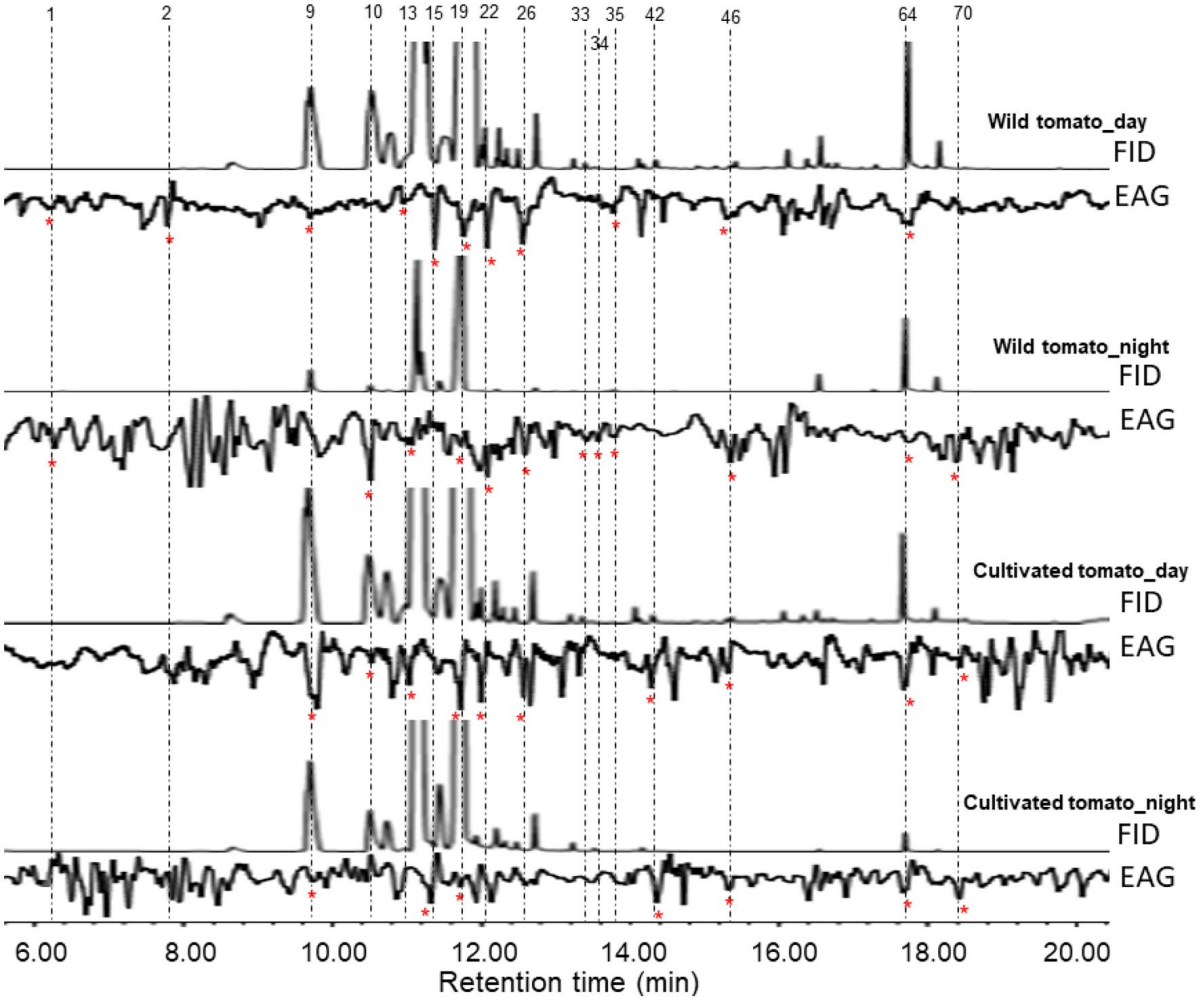
Profiles from the GC-EAG recordings. Numbers on top of the figure correspond to those listed in Table 1, and indicate compounds that elicited consistent antennal responses (marked with *) at least from three antennae from three insects. A total of 16 compounds were EAG-active, and listed as follows: 1 = hexanal; 2 = (*Z*)-3-hexenol; 9 = *α*-pinene, 10 = 3,7,7-Trimethyl-1,3,5-cycloheptatriene; 13 = *β*-myrcene; 15 = *α*-phellandrene, 19 = *β*-phellandrene, 22 = (*E*)-*β*-ocimene, 26 = terpinolene; 33 = limonene oxide; 34 = camphor; 35 = citronellal; 42 = methyl salicylate; 46 = *cis*-carvenone oxide; 64 = (*E*)-*β*-caryophyllene; and 70 = germacrene D.

The GC-EAD recordings demonstrated that a large proportion of the tomato headspace volatile constituents elicited electrophysiological responses of different intensities (Fig. 5). A total of 16 EAG-active compounds were found, including hexanal, (*Z*)-3-hexenol, *α*-pinene, *β*-myrcene, *α*-phellandrene, *β*-phellandrene, (*E*)-*β*-ocimene, terpinolene, limonene oxide, camphor, citronellal, methyl salicylate, (*E*)-*β*-caryophyllene (whose identification was confirmed using synthetic standards), and others tentatively identified as 3,7,7-Trimethyl-1,3,5-cycloheptatriene, germacrene D and *cis*-carvenone oxide. All compounds that elicited electrophysiological responses in the volatiles of the cultivated tomato plant were also detected by antennae of *T. absoluta* females in the volatiles of the wild tomato plant, except hexanal, (*Z*)-3-hexenol, limonene oxide and camphor which were unique to the headspace of the wild tomato plant (Table 1, Fig. 5).

### Wind tunnel bioassays using synthetic blends

**Figure 6:**
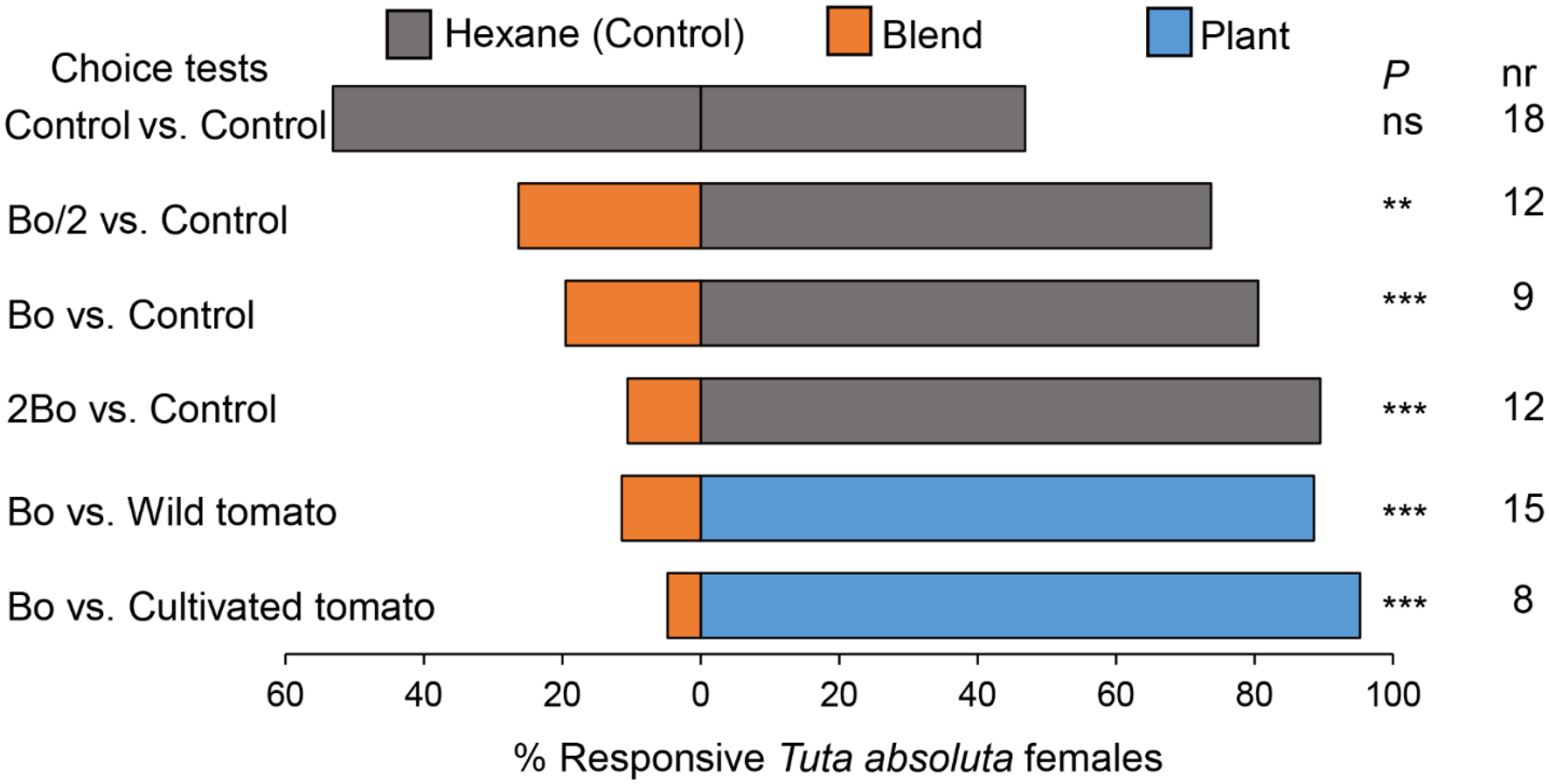
Behavioral responses (%) of *Tuta absoluta* females to synthetic blends and tomato volatiles. The original blend Bo is a 4-component blend tested at a dose containing 240 ng (*Z*)-3-hexenol, 240 ng limonene oxide, 280 ng citronellal and 400 ng camphor. Bo/2 and 2Bo are, respectively, half and double of the dose of the blend Bo. nr stands for the number of non-responsive insects (i.e., insects that made no choice) out of 50 insects tested per choice test. *P* stands for level of significance with ** and *** = significant difference at *P* < 0.01 and *P* < 0.001, respectively, from χ^2^ test at α = 0.05.

The blend of the 4 compounds [(*Z*)-3-hexenol, limonene oxide, camphor, and citronellal] unique to volatiles of the wild tomato plant showed dose-dependent repellence on mated *T. absoluta* females (Fig. 6). Significant numbers of *T. absoluta* avoided the dose of the original blend Bo (made of 240 ng (*Z*)-3-hexenol, 240 ng limonene oxide, 280 ng citronellal and 400 ng camphor), as well as the half and double of this dose compared to the control ((Bo/2): χ^2^ = 7.6, *P* < 0.01; (Bo): χ^2^ = 14.05, *P* < 0.001; (2Bo): χ^2^ = 22.13, *P* < 0.001) (Fig. 6). *Tuta absoluta* also showed significant levels of avoidance behavior to odor from the blend Bo compared to volatiles of the cultivated tomato plant (χ^2^ = 32.59, *P* < 0.001) or the wild tomato plant (χ^2^ = 19.03, *P* < 0.001) (Fig. 6).

## Discussion

We investigated the infestation rates of the tomato pinworm *T. absoluta* on wild and cultivated tomato plants in a field experiment, and characterized the volatiles mediating the avoidance behavior of the insect. Our findings indicated that the wild tomato, *S. lycopersicum* var. *cerasiforme,* is not preferred by *T. absoluta,* unlike the cultivated tomato, *Solanum lycopersicum* L. (var. Rambo F1). This is reflected in the reduced infestation levels recorded in the monocrop of the wild tomato plant compared to intercrops of the wild and cultivated tomato plants, which also had lower infestation levels compared to the monocrop of the cultivated tomato plant. When used as a border crop in the intercrops, the wild tomato plant was effective to reduce infestation by *T. absoluta* to the level reported in the monocrop of the wild tomato, indicating that it is enough to plant the wild tomato plant (as many as possible) around the cultivated tomato plant. Ghaderi et al.^26^ reported different field infestation levels by *T. absoluta* between monocrops of cultivated tomato cultivars, with the highest infestation level recorded on the cultivar ‘Cal JN3’ and the lowest on the cultivars ‘Early Urbana Y’ and ‘Super Strain B’. However, by the seventh week, there was a reduction of the larval infestation in the monocrop of cultivated tomato plants. The decrease in infestation could be explained by avoidance of tomato plants previously infested by conspecifics^28,29^, and older tomato plants by the moth^34^, as well as change in the volatile emission between phenological stages of tomato plants^35^. In our study, several factors including visual cues, trichome density, and headspace volatile composition could have contributed to the observed differences in the infestation levels between the wild and cultivated tomato plants. In our experiments, the infestation level was low on the plants, and it is worth replicating this study during a season of high infestation by *T. absoluta* in the field. Our preliminary behavioral tests in the wind tunnel showed that the cultivated tomato plant (var. Rambo F1) was found to be very attractive to gravid *T. absoluta* females, whereas the wild tomato plant (var. *cerasiforme*) was avoided by the moths. These observations are in agreement with the findings of Proffit et al.^27^ who reported that volatiles of the cultivated tomato (cv. Santa clara) were very attractive to females of *T. absoluta* while those of a wild tomato cultivar (*S. habrochaites*) were avoided by the insects. The “preference performance hypothesis” stipulates that insects will preferentially oviposit on host plants that guarantee the survival of their offsprings^36^. The avoidance of the wild tomato plant by *T. absoluta* females suggests that the offspring of the insect may not be able to complete development on the wild tomato plant, and it would be interesting to study the development of the larval stages of the pest on the wild tomato plant, and other tomato plants treated with extracts of the wild tomato. Dias et al.^37^ reported that *T. absoluta* females exhibited a reduced oviposition, and poor performance of larvae on the wild tomato *S. pennellii*, which was found to have high levels of acylsugars, unlike the cultivated tomato *S. lycopersicum* cv. Redenção’ which was attractive and suitable for the moth. Using biochemical and transcriptomic analyses, Chen et al.^38^ showed that the low level of infestation by *T. absoluta* on eggplant compared to tomato plant was attributed to specific signaling genes associated with emission of salicylic acid, and the high level of phenols in eggplant.

Allelochemicals play a major role in attracting or deterring gravid *T. absoluta* females, and in mediating their oviposition on host plants^27–29^. Our chemical analyses of the headspace of the tomato plants revealed quantitative and qualitative differences between the tomato plants and the collection times (day and night). Five compounds, namely limonene oxide, (*Z*)-3-hexenol, citronellal, camphor, and germacrene B were only detected in the volatile extract of the wild tomato (var*. cerasiforme*) and were not present in the volatile extract of the cultivated tomato (var. Rambo F1). However, Paudel et al.^39^ did not find any of these compounds in the volatiles of the wild tomato plant. The differences in the volatile profiles between our study and that of Paudel et al.^39^ could be explained by differences in the geographical regions (possibly different tomato accessions were used), as well as in the plant physiology and conditions of volatile collection, i.e., for Paudel et al.^39^, volatiles were collected at the vegetative stage in the laboratory, whereas in our study volatile collection was done in the field at the onset of flowering and fruiting stage. Among compounds found to be specific to the wild tomato (var*. cerasiforme*), germacrene B was reported in the wild tomato *S. habrochaites*^27^, and (*Z*)-3-hexenol, citronellal and limonene oxide were reported in *S. pimpinellifolium*^40^ which is considered to be a parental line of the wild tomato (var*. cerasiforme*)^41^. In this study, we found that the tomato plants emitted more volatiles during the day than at night. The monoterpenes tricyclene, *α*-terpineol and thymol, the ketones (–)-car-3-en-2-one and *p*-ethyl acetophenone, the aldehyde *α*-terpinen-7-al, the alcohol *p*-cymen-7-ol, and the sesquiterpenes (*Z*)-*β*-caryophyllene and *α*-cedrene were detected in the day volatiles of both wild and cultivated tomato plants but these compounds were absent in the night volatile extracts (Table 1). The differences between day and night volatile compositions could be explained by differences in the physiological responses of the plants when exposed to environmental stresses. In our experiment, the temperature at night was 20 ± 3 °C, slightly lower than that of the day, 25 ± 3 °C. It has been reported that tomato plants exhibit a lower degree of transpiration and partial stomata opening during low night temperatures than at high day temperatures^42,43^, and these are among the factors that have been reported to explain higher volatile emission in the day than at night^44^. Irrespective of the collection times, *β*-phellandrene and *δ*-2-carene were the most abundant monoterpenes, while (*E*)-*β*-caryophyllene and *α-*humulene were the most abundant sesquiterpenes, which are in agreement with a previous study on volatiles emitted by the wild tomato (var. *cerasiforme*)^39^, as well as cultivated tomato cultivars like Moneymaker^45^, Semiramis^29^, and Kilele F1^33^. We found that the total emissions of volatiles did not significantly differ between the wild and cultivated tomato plants during the day or at night, but quantitative differences were noted in the emission of some compounds between the tomato genotypes. The emission level of *α*-thujene was higher in the wild tomato plant than in the cultivated, whereas emission levels of tricyclene and *β*-pinene were higher in the cultivated than in the wild tomato plant.

The use of multivariate tools (machine learning) for the identification of discriminating volatiles between treatments is gaining attention in the field of chemical ecology^46,47^. Several of these variable selection methods including the random forest (RF), the sparse partial least square discriminant analysis (sPLS-DA) and the non-metric multidimensional scaling (NMDS) have been used in the literature to narrow down important volatiles for use in behavioral assays to determine bioactive compounds^33,48,49^. We found that volatile compounds selected as important for discriminating the volatile extracts of the wild and cultivated tomato plants varied with the multivariate method used (Fig. 3a–c); hence a combination of methods could provide a better discrimination. Similarities in the top 10 selected discriminating compounds were common when using the mean decrease in accuracy of RF and the variable importance in the projection of the sPLSDA, unlike for the NMDS. In our study, the top 10 discriminating VOCs predicted by RF and sPLS-DA were either minor (e.g., (*Z*)-3-hexenol and *β*-pinene), absent in some volatile treatments (e.g., limonene oxide, citronellal, camphor and germacrene B unique to the wild tomato plant), or significantly different among the treatments irrespective of the volatile amount (e.g., *δ*-3-carene and *α*-humulene). Our observations are consistent with findings reported by Peterson et al.^49^ who used RF to highlight compounds that distinguished volatiles of three plant species. On the contrary, we observed that NMDS was tuned to select compounds that are abundant in the volatile treatments, even at levels not significantly different between the treatments. Using NMDS to select distinguishing volatiles between six *Lygodium* plant species, Wheeler et al.^50^ also found that the top 6 discriminating VOCs were the major constituents emitted by the plants. The observed differences in the discriminating compounds selected by the marching learning methods could be explained by differences in assumptions and algorithms that composed each method^51^. When looking at compounds that best discriminate headspace volatile composition and those that could potentially play a role in the behavioral responses of insects, a single variable selection method is unlikely to predict most of the bioactive compounds. We found 16 compounds (i.e., hexanal, (*Z*)-3-hexenol, *α*-pinene, 3,7,7-Trimethyl-1,3,5-cycloheptatriene, *β*-myrcene, *α*-phellandrene, *β*-phellandrene, (*E*)-*β*-ocimene, terpinolene, methyl salicylate, (*E*)-*β*-caryophyllene, germacrene D and *cis*-carvenone oxide) that were detected by antennae of *T. absoluta* females. Among these compounds, five (i.e., (*Z*)-3-hexenol, limonene oxide, camphor, citronellal and (*E*)-*β*-caryophyllene) were present in the top 10 discriminating VOCs highlighted by RF or sPLSDA, while other four compounds (*α*-pinene, 3,7,7-Trimethyl-1,3,5-cycloheptatriene, *α*-phellandrene and *β*-phellandrene) were predicted by NMDS. Therefore, a combined use of variable selection methods could provide a better comparison of discrimination between different headspace volatiles and increase the chance of discovering more bioactive compounds, although this would imply testing several compounds, which might also be tedious.

*Tuta absoluta* females are sensitive to small variations in headspace compositions emanating from different tomato varieties and cultivars, as well as other host plants, which led to different levels of attraction or repellency^27–29,52^. The availability of several overlapping bioactive compounds in the headspace volatiles of host plants has been associated with the polyphagy nature of the herbivorous pest^53^. Among the EAG-active compounds reported in this study, antennae of *T. absoluta* females were found to detect synthetic standards of hexanal, (*Ζ*)-3-hexenol, *α*-pinene, *α*-phellandrene, *β*-myrcene and methyl salicylate^29^ and be attracted to a blend of five compounds among them *β*-ocimene, and (*E*)-*β*-caryophyllene^53^. To the best of our knowledge, camphor, citronellal, limonene oxide, *β*-phellandrene, terpinolene, (*E*)-*β*-ocimene, (*E*)-*β*-caryophyllene, and others tentatively identified as 3,7,7-Trimethyl-1,3,5-cycloheptatriene, germacrene D and *cis*-carvenone oxide were not reported as EAG-active compounds for *T. absoluta*. In future research, it is important to run a mixture of synthetic standards of these compounds in GC-EAD recordings to confirm their detection by the insect’s antennae. Although the wild and cultivated tomato plants had opposite effects on the responses of *T. absoluta* females, most of the EAG-active compounds were commonly emitted by both plants. Hence, it is likely that the EAG-active compounds that mediated the observed avoidance behavior in *T. absoluta* females to the wild tomato were those which were present only in volatiles of the wild tomato. Interestingly, we found that the 4-component blend of the EAG-active compounds [(*Z*)-3-hexenol, camphor, citronellal and limonene oxide] which were uniquely emitted by the wild tomato plant elicited avoidance behavior in *T. absoluta* females in a dose-dependent way when compared to control solvent. Moreover, the blend repelled the insects when compared to volatiles of the wild or cultivated tomato plant. *Tuta absoluta* is a nocturnal moth, hence the insect could be more sensitive to volatiles emitted at night. Except for (*Z*)-3-hexenol which was emitted at a higher level during the day than at night, the other three compounds of the blend were released in higher amounts at night. Steen et al.^54^ also reported that the optimal foraging activity by the nocturnal moth, the pine hawkmoth *Sphinx pinastri* L. (Lepidopera: Sphingidae) to *Platanthera chlorantha* plant coincided to the period of increase in the night emission of (*E*)-*β*-ocimene and (*Z*)-*β*-ocimene.

Natural enemies and insect pests may detect the same VOCs from host plants, which can turn out to be of advantage or disadvantage for the control of insect pests when using allelochemicals. Among the compounds that are detectable by antennae of *T. absoluta*, some *T. absoluta* parasitoids like *Trichogramma cordubense* Vagas & Cabello and *Trichogramma achaeae* Nagaraja & Nagarkatti (Hymenoptera: Trichogrammatidae) were reported to detect *β*-myrcene and (*Z*)-3-hexenol^55^, and *Trichogramma chilonis* Ishii (Hymenoptera: Trichogrammatidae) was found to detect hexanal, citronellal, (*Z*)*-*3*-*hexenol, *α*-pinene, *α*-phellandrene and (*E*)-*β*-caryophyllene in EAG recordings^56^. Moreover, De-Backer et al.^57^ reported that antennae of the predator *Macrolophus pygmaeus* (Rambur) (Hemiptera: Miridae) detect hexanal, *α*-pinene, and *β*-phellandrene. In olfactometer bioassays, other natural enemies of *T. absoluta* such as the parasitoid *Dolichogenidea gelechiidivoris* (March) (Hymenoptera: Braconidae) were reported to be attracted to *α*-pinene, *α*-phellandrene, *β*-ocimene, methyl salicylate and (*E*)-*β*-caryophyllene^33^, and the predator *Nesidiocoris tenuis* (Reuter) (Hemiptera: Miridae) to *α*-pinene, *α*-phellandrene, *β*-ocimene and *δ*-3-carene^58^, and to (*Z*)*-*3*-*hexenol and methyl salicylate^59^. Furthermore, some of the compounds attractive to the natural enemies were emitted in high amounts by the wild tomato plant, suggesting that the use of this tomato in intercropping systems could play a double beneficial role in the control of *T. absoluta*, through the attraction of the natural enemies and deterrence of oviposition by the moth. The possible effect of the wild tomato plant, and the 4-component blend (mixture of (*Z*)-3-hexenol, camphor, citronellal and limonene oxide) repellent to *T. absoluta* with the inclusion of other EAG-active compounds, on the behavioral responses of these natural enemies is worth evaluating in future research.

In summary, our findings show that the level of infestation by *T. absoluta* was lower on the wild tomato *S. lycopersicum* var. *cerasiforme* compared to that on the cultivated tomato *S. lycopersicum* (var. Rambo F1). Chemical analyses reveal both qualitative and quantitative differences in the volatile compositions of the wild and cultivated tomato plants. A total of 16 compounds were detected by antennae of *T. absoluta* females. Among these EAG-active compounds, the 4-component blend of (*Z*)-3-hexenol, camphor, citronellal, and limonene oxide identified only in volatiles of the wild tomato plant showed dose-dependence repellency to *T. absoluta* females. The present study lays down some groundwork for the development of a ‘push–pull’ strategy to control *T. absoluta* through intercrops of wild tomato with cultivated tomato plants, and deployment of traps or agro-nets baited with optimized controlled-release of the repellent blend*.*

## Materials and methods

### Wild and cultivated tomato plants

Seeds of the cultivated tomato *Solanum lycopersicum* L. (var. Rambo F1) were purchased from Angro-Pest Fighter shop (Royal Seed Company, Kenya) while the seeds of the wild tomato, *Solanum lycopersicum* L. var. *cerasiforme* (locally referred to as *Kinyanya*) were provided by a farmer at the Kenyan Agricultural and Livestock Research Organization (KALRO), Kirinyaga County, Kenya. This wild tomato is commonly known as the wild form of cherry tomato, and is considered to be an evolutionary intermediate between the domesticated or cultivated tomato *S. lycopersicum* and its wild ancestor, *S. pimpinellifolium*^41,60^. Seedlings of the two tomato genotypes were grown in nurseries using a mixture of soil and manure (goat and chicken dungs) in the ratio of 3:1, at Wang’uru in Mwea planes (S 00′4137.4″; E 037°22′12.3″), Kirinyaga County, Kenya. After four weeks in the nurseries, seedlings of each plant were transplanted on the mixture of soil and manure in the open field with the addition of 15 g of diammonium phosphate (DAP) at planting. These plants were used to assess the effects of monocropping and intercropping of the wild and cultivated tomato plants on infestation levels of *T. absoluta*. Other seedlings of each tomato were transplanted in a screened nethouse to protect them from herbivorous pests, and these plants were thereafter used for volatile collection in the field. Watering was done twice per week. After 3 weeks, a top dressing with 15 g of calcium ammonium nitrate was done for each plant.

### *Tuta absoluta* colony

*Tuta absoluta* adults were obtained from the insectary at *icipe* (S01°13.140′; E036°53.440′), Nairobi, Kenya, which was established in early 2015 and supplemented regularly with wild adults that emerged from larvae-infested tomato plants collected from open field plantations. Adult males and females of *T. absoluta* were placed in Perspex cages (65 cm × 45 cm × 45 cm) and fed with a mixture of 80% honey and water. The colony was maintained under controlled laboratory conditions at a temperature range of 25 ± 2 °C and 70 ± 5% RH and a cycle photoperiod of 12:12 h L:D. Potted plants of the cultivated tomato were exposed to *T. absoluta* adults for 3 days for oviposition, and then the plants with eggs were transferred to other cages where the eggs hatched. The larvae which developed were offered additional plants to ensure better development through to the pupation stage. Mated *T. absoluta* females of 3-days old were used in the bioassays. To ensure that gravid females were used in the behavioral assays, couples that were mating in the rearing cage were aspirated and put into individual cages (10 cm × 10 cm × 6 cm). Thereafter, females were separated from males based on differences in their body morphology (i.e., females are larger with a broader abdomen tip than the males who have a slender abdomen tip).

### Field layout for assessing relative infestations of *Tuta absoluta* in monocrops and intercrops of the two tomato genotypes

A 4-factor randomized block design (RBD) was used on a 1/4-acre piece of land divided into 16 plots (4 m × 4 m each). Four treatments were set up, namely, monocrop of the wild tomato (WT), monocrop of cultivated tomato (CT), intercrops of the two (CT + WT), and monocrop of cultivated tomato surrounded by one raw of the wild tomato serving as a border [WT (CT)], and these were arranged in four blocks. Thirty-six seedlings were transplanted 75 cm apart on each plot (6 rows and 6 columns), and 4 m separated one plot from another. In the intercrops of the two (CT + WT), a cultivated tomato plant was alternated with a wild tomato plant, making 18 individual plants of each tomato genotype per plot. In the plot where the wild tomato plant was used as a border (WT(CT)), 20 individuals of the wild tomato plant surrounded 16 individuals of the cultivated tomato plant. Three weeks after transplanting, forty leaves were randomly sampled from 20 plants (two leaves per plant) per plot in the monocrops, and from 15 cultivated plants and five wild plants in the intercrops. The sampled leaves were put into labeled polythene bags, put into a cool box, then transported to the laboratory at *icipe* for visual observation and counting of *T. absoluta* mines and larvae under a glass magnifier. This process was replicated every week for 7 weeks. The plant experiments in the field were performed following relevant guidelines and regulations by the National Commission for Science, Technology and Innovation (NACOSTI), Nairobi, Kenya (Ref.: NACOSTI/RCD/ST& I7th CALL/ M.Sc/037).

### Trapping of headspace volatiles from naturally growing tomato plants

The volatile collection was done at a field station at Wang’uru in Mwea planes (S 00′4137.4″; E 037°22′12.3″), Kirinyaga County, Kenya, where the plants were grown in their natural habitats but screened from herbivorous insect pests using fine size mesh nets. A dynamic push–pull headspace trapping technique was used in the collection of volatiles from the two different tomato cultivars. Six-week-old plants were used for volatile collection in situ under nethouse in the field (Supplementary Fig. S1). The aerial parts (with leaves, flowers and young fruits) of a single plant were enclosed in polyacetate oven bags (25 cm × 38 cm) (KitchenCraft, Thomas Plant (Birmingham) Ltd, UK) that were connected to clean air supplied by a vacuum pump packed with a charcoal column filter (Analytical Research system Inc., Gainesville, FL, USA) (Supplementary Fig. S1). The bags were kept in an oven at ~ 100 °C for 24 h before use. Clean air from the activated charcoal-based filter entered the bags at a rate of 250 mL/min and volatiles were trapped in 30 mg Super-Q absorbent (ethyl vinyl benzene–divinylbenzene polymer) traps (Analytical Research System, Gainesville, FL, USA). Four replicates were collected from each tomato variety and control (bag without plant) for 8 h during the day and at night. Day and night temperatures were recorded and were as follows: 25 ± 3 °C and 20 ± 3 °C, respectively, and relative humidity of 68 ± 17% and 70 ± 19%, respectively. Volatiles collected during the day and night were henceforth referred to as CTDV (cultivated tomato day volatiles), WTDV (wild tomato day volatiles), CTNV (cultivated tomato night volatiles), and WTNV (wild tomato night volatiles). The super-Q traps containing the headspace volatiles were first sealed using 0.075 mm P.T.F.E. thread seal tape (MAAT, UK), then lapped in aluminum foil, and put in a cool box containing dry ice (Carbacid (CO_2_) Limited) (Carbacid Investment Limited, Nairobi, Kenya), after which the samples were transported to the laboratories at *icipe* for elution. Elution of each volatile replicate was done under controlled laboratory conditions using 200 μL of dichloromethane (DCM) into a 2 mL glass vial under a gentle stream of high purity nitrogen used as the eluting gas. The eluents were stored at − 80 °C until use.

### Analyses of tomato headspace volatiles

Analyses of headspace volatiles from wild and cultivated tomato plants were done using GC–MS, a 7890A gas chromatography coupled to a mass selective detector (Agilent Technologies, Palo Alto, California, USA) equipped with an HP-5MS column of 30 m length × 0.25 mm i.d. × 0.25 µm film thickness (Agilent technologies, Palo Alto, California, USA). One microlitre (1 µL) aliquot of headspace volatile extract collected during the day and at night from each tomato was injected into the GC and analyzed in splitless mode. Helium was used as the carrier gas at a flow rate of 1.2 mL/min. The initial oven temperature was set at 35 °C and held for 5 min, then programmed to increase at 10 °C/min to 280 °C which was maintained for 10.5 min. Compounds from the headspace of tomato plants (see Supplementary Fig. S2 for representatives of the GC–MS profiles) were tentatively identified using retention times, electron ionization spectrum, and Kovats retention indices, comparison of their spectra with the library data (Adams2, Chemecol, and NIST11), as well as comparison with published Kovats retention indices and mass spectra from online NIST library database. Available authentic standards were run (Supplementary Fig. S3) to confirm some of the identified compounds based on retention time and mass spectra. The mass spectra of unidentified compounds are provided in supplementary materials (Fig. S4). Each compound detected in the headspace volatiles was quantified using the external calibration method. Due to unavailability of synthetic standard from each reported chemical class, a relative quantification was performed using the linear equation of a monoterpene to quantify compounds with a retention time of less than 16 min (range where monoterpenes are dominant in the headspace volatiles), and the linear equation of a sesquiterpene to quantify compounds with a retention time beyond 16 min, where sesquiterpenes are dominant in the headspace volatiles, and this followed the quantification method used by Njuguna et al.^61^. The monoterpene *α*-pinene and the sesquiterpene *α*-humulene were run at different concentrations and the linear equations were generated from the calibration curves (Supplementary Fig. S5). Thereafter, the means of the release rates (ng/µL/h) of the compounds were computed.

### Gas chromatography-linked electroantennographic detection (GC-EAD)

Hewlett-Packard (HP) 5890 Series II gas chromatograph equipped with an HP-5MS column (30 m × 0.25 mm i.d. × 0.25 µm film thickness, Agilent Technologies, Palo Alto, California, USA) was used with high purity nitrogen as the carrier gas at a flow rate of 1 mL/min flow. Four microlitres (4 µL) aliquot of each volatile extract was injected into the entry port of the GC-FID at an injection temperature of 280 °C and a split valve delay of 5 min. The oven temperature was held at 35 °C for 3 min, then programmed to increase at 10 °C/min to reach 280 °C and maintained at this temperature for 10 min. The column effluent was split 1:1 for simultaneous detection by the flame ionization detector (FID) and electroantennographic detection (EAD). For EAD, silver-coated wires in drawn-out glass capillaries (1.5 mm i.d.) were filled with Ringer saline solution (7.5 g NaCl, 0.7 g KCl, 0.2 g CaCl_2,_ and 0.2 g MgCl_2_ dissolved in 1L of distilled water). Antennal preparations were made by putting the gravid *T. absoluta* female in a glass vial which was placed on ice for 10 min to immobilize the insect, after which the base of the head and distal end of the antennae were cut off with a scalpel. The base of the head was then connected to the reference electrode while the tip of one antenna was connected to the recording electrode. The analog signal was detected through a probe (INR-II, Syntech, Hilversum, the Netherlands), captured and processed with a data acquisition controller (IDAC-4, Syntech, the Netherlands), and later analyzed with software (EAG 2000, Syntech). Each plant volatile extract (i.e., cultivated tomato plant day volatiles, cultivated tomato plant night volatiles, wild tomato plant day volatiles, and wild tomato plant night volatiles) was analyzed 6 to 10 times using a fresh female antenna at each run, making a total of between 24 and 40 antennae tested in the GC-EAD recordings. The EAG recordings were checked, and 4 replicates were selected per each headspace volatile, then every EAG response that matched with a compound was screened, and only compounds that elicited three consistent EAG responses, i.e., compounds detected by at least 3 antennae, were considered as EAG-active compounds used in the results.

### Behavioural responses of gravid *Tuta absoluta* females to synthetics of electrophysiologically-active compounds

The responses of mated *T. absoluta* females to a blend of synthetic compounds were observed in a dual-choice cuboidal, Perspex glass wind-tunnel (150 cm × 21 cm × 21 cm) (Supplementary Fig. S6). Each end of the wind tunnel was connected to a 250 mL quick-fit glass jar (Sigma Scientific, Gainesville, FL, USA) serving as an odor source container, using flexible PTFE tubes (Supplementary Fig. S6). Ambient air was sucked using a vacuum pump (Analytical Research system Inc., Gainesville, FL, USA), then passed through an activated charcoal column filter and a water chamber respectively for filtering and moisturization. The air-flow rate was set at 350 mL/min in each arm of the wind tunnel using a flowmeter (AALBORG, Orangeburg, NY, USA), and was pulled from the bioassay set-up at a rate of 700 mL/min through the centre of the tunnel. A red fluorescent tube was suspended 1.5 m above the tunnel giving about 1000 lx incident red light under which behavioral observations were performed to prevent the insects from using visual cues when making choices. The bottom of the tunnel was marked every 5 cm from the centre to the two ends to facilitate the measurement of the insects’ flight distances.

Among the consistent EAG-active compounds which were unique to the wild tomato, an original blend Bo made of (*Z*)-3-hexenol, limonene oxide, camphor, and citronellal was composed using a tenfold of the compound release rates mixed in ratio found in the volatile emission of the wild tomato (Table 1). Specifically, the blend Bo contained 1.2 ng/µL (*Z*)-3-hexenol, 1.2 ng/µL limonene oxide, 1.4 ng/µL citronellal and 2 ng/µL camphor. Thereafter, the concentration of the blend Bo was halved (Bo/2) and doubled (2Bo) to make two additional blends. The blends were prepared in hexane used as the solvent*.* An aliquot of 200 µL of the blends (treatments) or the solvent (control) was applied onto a 1 cm long Luna dental roll (Roeko^®^, Langenau, Germany). Hence, the dose tested for the original blend Bo contained 240 ng (*Z*)-3-hexenol, 240 ng limonene oxide, 280 ng citronellal and 400 ng camphor. The following dual choices were tested: (i) pure hexane versus pure hexane (control test), (ii) blend Bo dose versus the control (pure hexane), (iii) half of blend Bo dose (Bo/2) versus the control, (iv) double of Bo dose (2Bo) versus the control, (v) blend Bo dose versus volatiles of the cultivated tomato, and (vi) blend Bo dose versus volatiles of the wild tomato. The impregnated dental rolls were left for 20 min at room temperature to enable evaporation of the solvent after which the odor sources (i.e., the dental roll impregnated with solvent or volatile blend, or the healthy plant) were immediately introduced into the odor containers (i.e., 250 mL quick-fit jar for the volatile blend, and a cuboidal Plexiglass cage (61 cm × 35 cm × 35 cm) for the plant). Ten minutes later, a group of ten (10) mated *T. absoluta* females were released through a hole made at the centre of the wind tunnel. The released insects were observed for 20 min, and the numbers of responsive individuals were recorded. *Tuta absoluta* females that landed beyond 35 cm of either side away from the centre were considered to have responded to the corresponding odor or the control; whereas insects that remained between the release point and 35 cm of either side were considered as non-respondents (Supplementary Fig. S6) which were thereafter not included in the data analysis. After each run of ten insects, air flow was allowed to pass through the wind tunnel set-up for 1 h to remove the previous test odor, then odor sources and containers were renewed, and locations of the containers switched between left and right of the wind tunnel. Each assay was replicated five times on 5 days, with a group of ten insects tested per day per choice test, making a total of 50 insects tested per choice test. The odor containers were cleaned with hot water and Teepol odourless detergent, rinsed with distilled water, then dried in the oven at 150 °C overnight before they were used again on another day. The bioassays were conducted at 23 ± 2 °C and 65 ± 5% RH and between 0500 to 1100 h AM as preliminary observations in the laboratory had indicated this period as the optimal activity time for the insect.

### Chemicals

Synthetic standards used in the bioassays were: (*Z*)-3-hexenol, limonene oxide, camphor and citronellal. Other standards used for the confirmation of some of the identified compounds included*,* hexanal, *α*-pinene, *β*-pinene, *p*-xylene, *p*-cymene, *β*-myrcene, *δ*-2-carene, *δ*-3-carene, methyl salicylate, decanal, *α*-phellandrene, *α*-terpinene, *β*-phellandrene, (*E*)-*β*-caryophyllene, *β*-ocimene [mixture of (*E*) and (*Z*)], *α*-terpineol, linalool, terpinolene, nonanal, allo-ocimene, *γ*-terpinene, *β*-elemene, and *α*-humulene. The chemical purity was between 90–99%, except for *α*-phellandrene (85%) and *α*-terpinene (85%). All these standards were purchased from Sigma-Aldrich, Germany. Dichloromethane (99.9% purity) used for volatile elution was purchased from Merck, Germany. Hexane (98%) used as the solvent for blend preparation was purchased from Sigma-Aldrich, Germany.

### Statistical analyses

All data analyzed in this study were collected between 2015–2016. All statistical analyses were performed using R statistical software, version 4.0.2^62^. The data on infestation levels and volatile release rates were first subjected to test for normality using Shapiro–Wilk’s test, and homogeneity of variance using Bartlett’s test. The data on infestation levels were auto-scaled using log_10_χ + 1 and then subjected to a one-way analysis of variance (ANOVA) to compare the mean numbers of mines and larvae per week per leaf, which was followed by Student Neuman Keuls (SNK) test to separate means when a significant difference was noted. Thereafter, the data were averaged per treatment level and ANOVA was performed to find the effects of the treatments on the numbers of mines and larvae throughout the experimental period. The data on volatile release rates were analyzed using a non-parametric Kruskal–Wallis ANOVA to compare amounts of VOCs between day and night volatiles of the cultivated and wild tomato plants, and when a significant difference was noted a post hoc Dunn’s test with Bonferroni’s adjustment was applied for mean separation^63^. Based on the release rates, the VOCs that best distinguished the wild tomato from the cultivated tomato were screened using a combination of machine learning variable selection tools, e.g., the mean decrease in accuracy (MDA) of the random forest (RF) analysis^64^, the variable importance in the projection (VIP) of the sparse partial least square discriminant analysis (sPLS-DA)^65^, and the similarity percentage (SIMPER) of the non-metric multidimensional scaling (NMDS)^66^. Compounds that appeared in the top 10 of the VOCs highlighted by these three variable selection methods were considered as the most discriminating VOCs between day and night volatiles of the wild and cultivated tomato plants. Using the release rates of the most discriminating VOCs, biplots of sPLS-DA and NMDS were performed, respectively, in the mixOmics package and Past Software, to illustrate the correlation between volatile compounds and the tomato plants^65^. A multidimensional scaling (MDS) plot was also performed to visualize the similarity among volatiles of the plants using the function “MDSplot” of the RF package^67^. A clustering heatmap was done using the function “cim” in the mixOmics package^68^ to illustrate variations in the discriminating VOCs across replicates of day and night volatile extracts of the tomato plants. The out-of-bag (OOB) error of the RF analysis was used to appreciate the classification accuracy (100%—OOB error) by RF^64^. The sPLS-DA model was validated using the function “perf” and the parameters (Q2, R2X, R2Y), as well as the “leave-one-group-out” cross-validation method in the mixOmics package^68^. One-way analysis of similarities (ANOSIM) associated with the Bray–Curtis dissimilarity index was performed to appreciate the validity of the NMDS model^66^. For the behavioral assays, the frequencies of choice made by *T. absoluta* females in the wind tunnel were compared between the odors using a Chi-square (χ^2^) goodness of fit test.

## Supplementary Information

Supplementary Figures.

## Data Availability

Data generated and analyzed in this study are available on request from the corresponding authors.
