# Supplementary material for: Electroantennogram and machine learning reveal a volatile blend mediating avoidance behavior by Tuta absoluta females to a wild tomato plant
Source: Sci Rep. 2022 May 27;12:8965. doi: 10.1038/s41598-022-13125-0 (PMC9142488; doi:10.1038/s41598-022-13125-0)
Supplement: Supplementary file 1 — Supplementary Figures. [file 41598_2022_13125_MOESM1_ESM.pdf]

TITTLE: Electroantennogram and machine learning reveal a volatile blend mediating avoidance behavior by *Tuta absoluta* females to a wild tomato plant

AUTHRORS

Raphael Njurai Miano[1,2*], Pascal Mahukpe Ayelo[1], Richard Musau[2], Ahmed Hassanali[2] & Samira A. Mohamed[1*]

[1]International Centre for Insect Physiology and Ecology (*icipe*), P.O Box 30772-00100, Nairobi, Kenya

[2]Department of Chemistry, Kenyatta University, P.O Box 43844-00100, Nairobi, Kenya

***Correspondence to rmiano@icipe.org ; sfaris@icipe.org**

Supplementary materials

Supplementary Figure 1

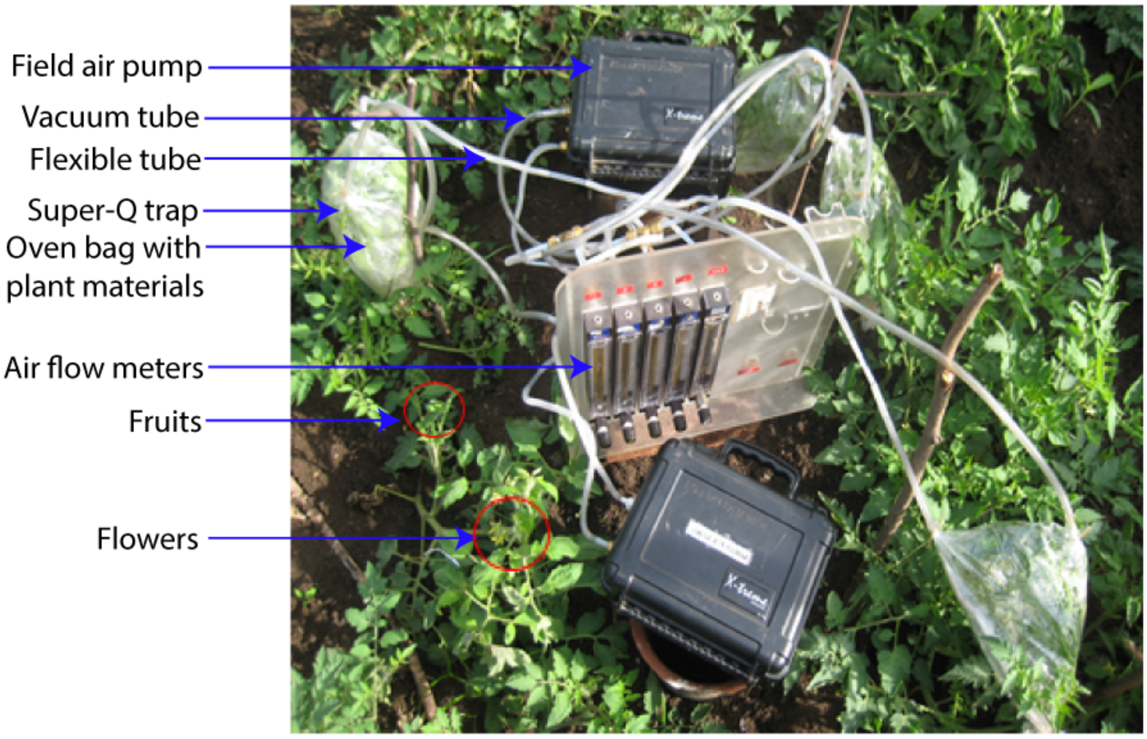

Figure S1 Set-up for the volatile trappings in the field.

Supplementary Figure 2

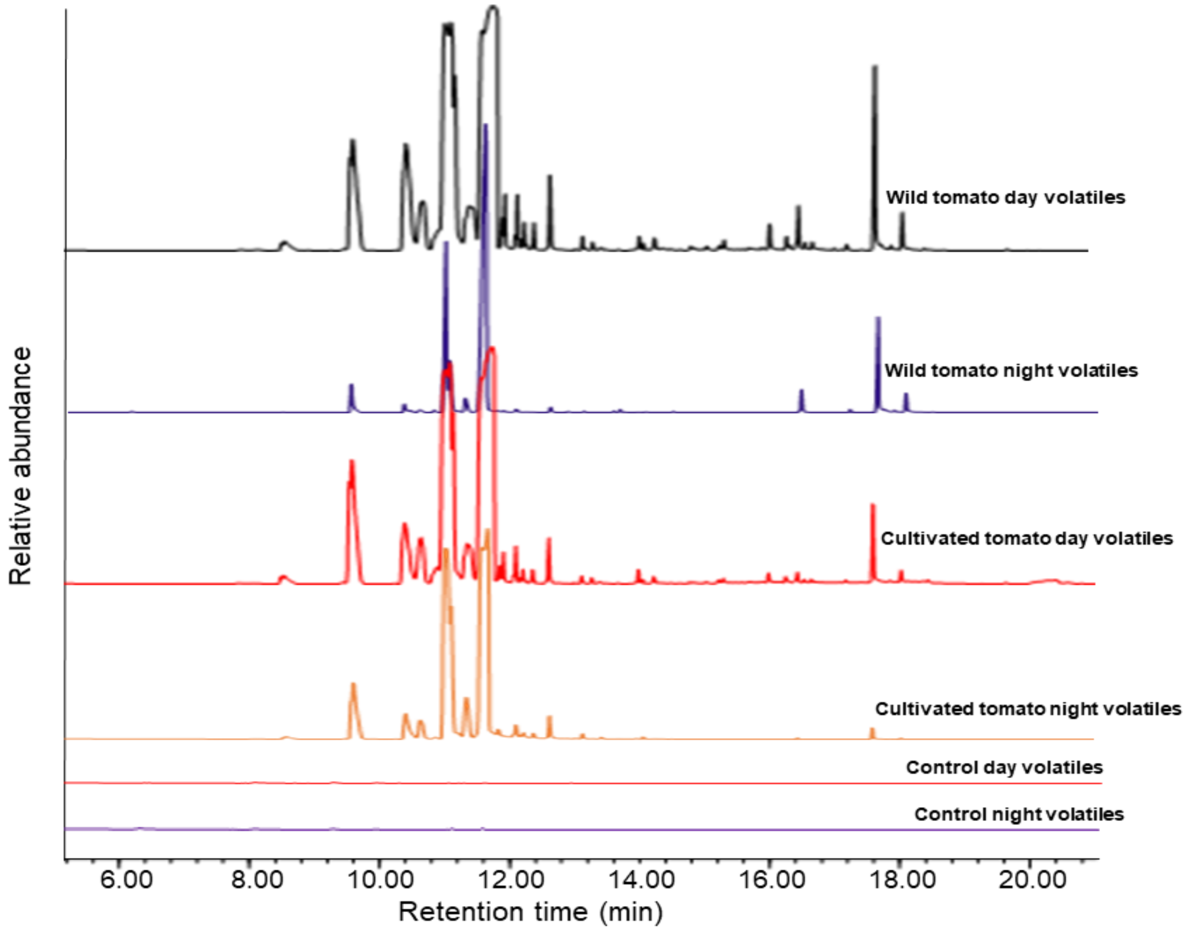

Figure S2 Representatives of GC-MS profiles of the headspace volatiles collected during the day and at night from the wild (var. *cerasiforme*) and cultivated (var. Rambo F1) tomato plants.

Supplementary Figure 3

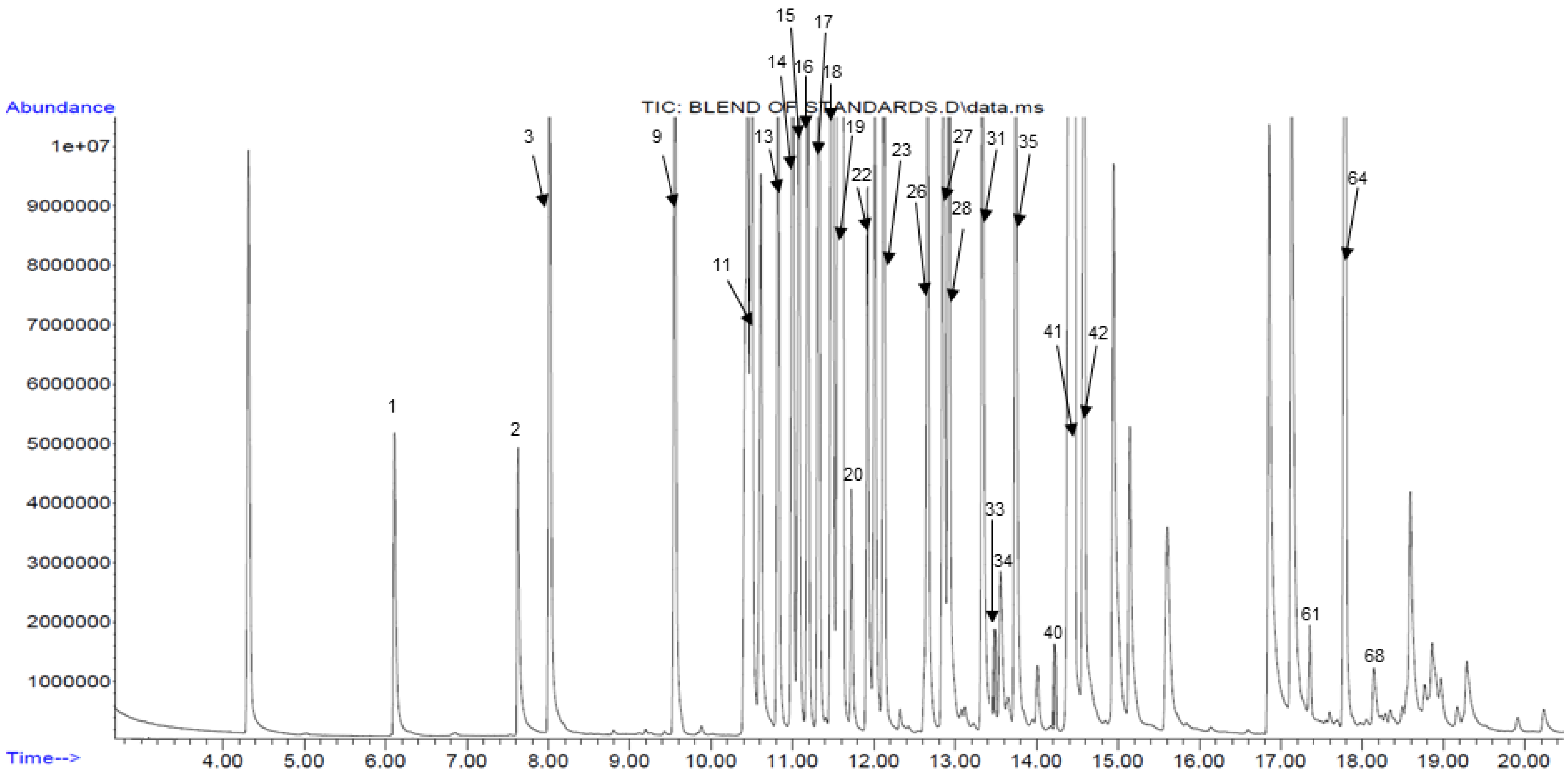

Figure S3 Chromatogram of the chemical standards run to confirm some of the identified compounds. Numbers correspond to names of compounds listed in Table 1.

Supplementary Figure 4

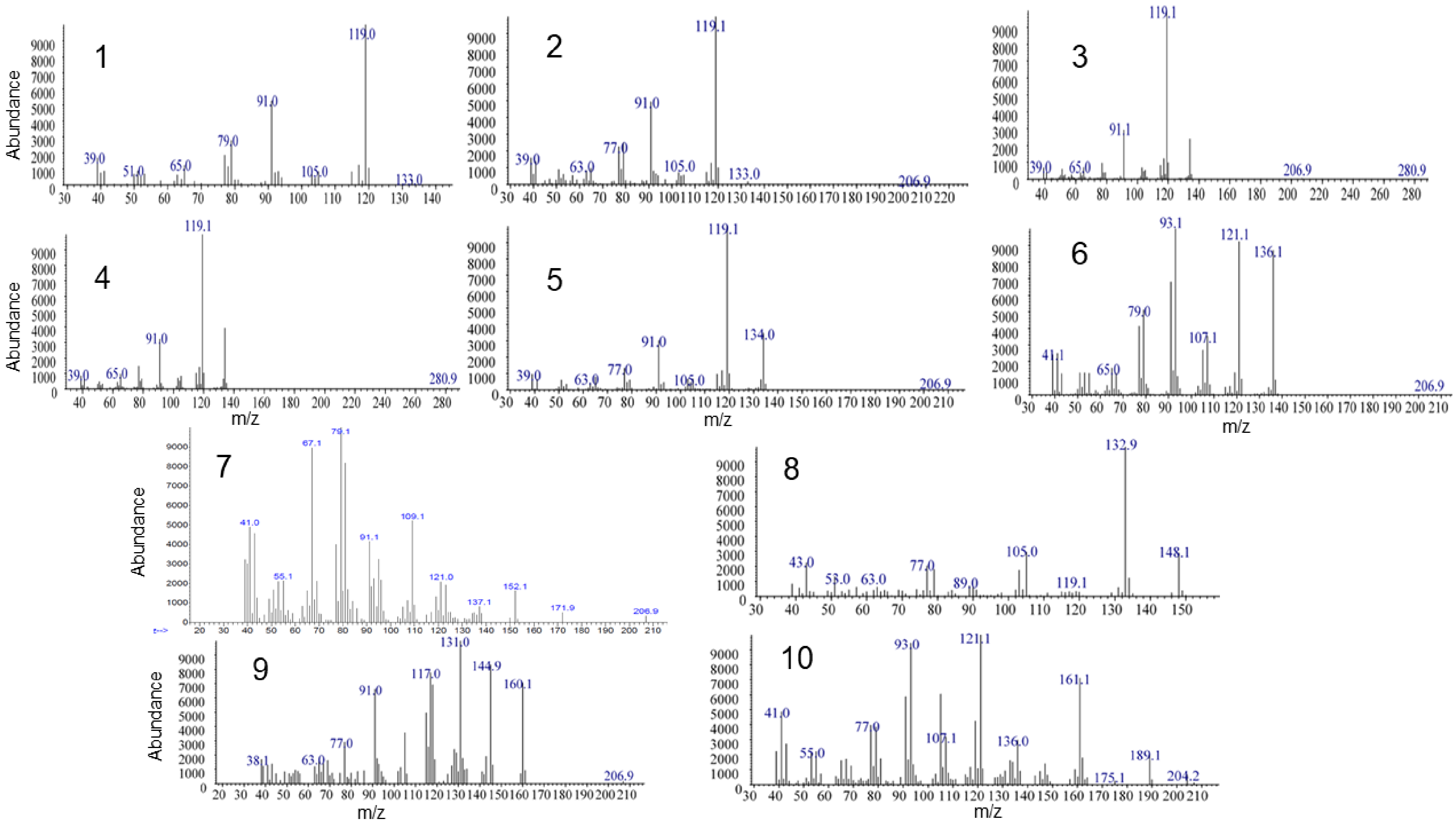

Figure S4 Mass spectra of the ten unidentified compounds numbered from 1 to 10, as listed in table 1.

Supplementary Figure 5

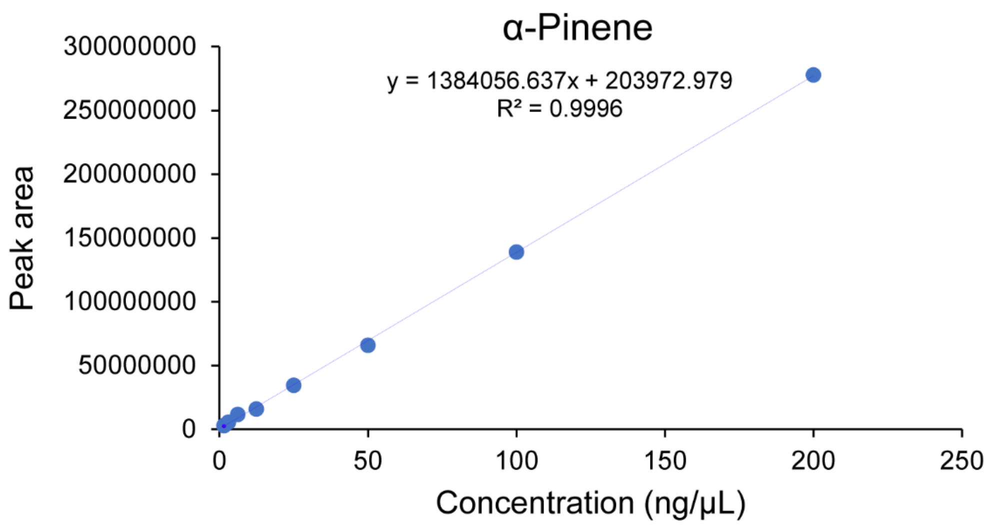

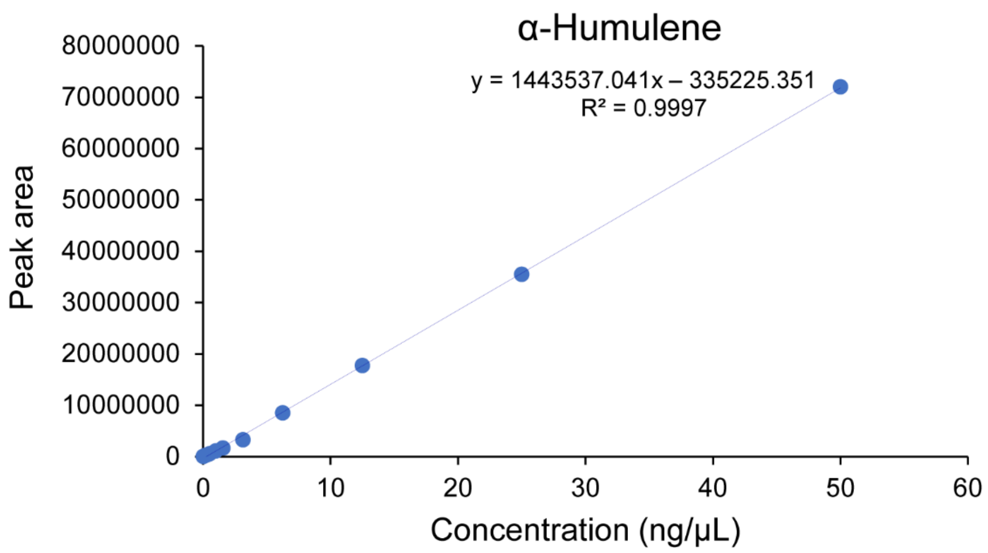

Figure S5 Calibration curves showing the linear equations used in the quantification of the identified volatiles compounds.

Supplementary Figure 6

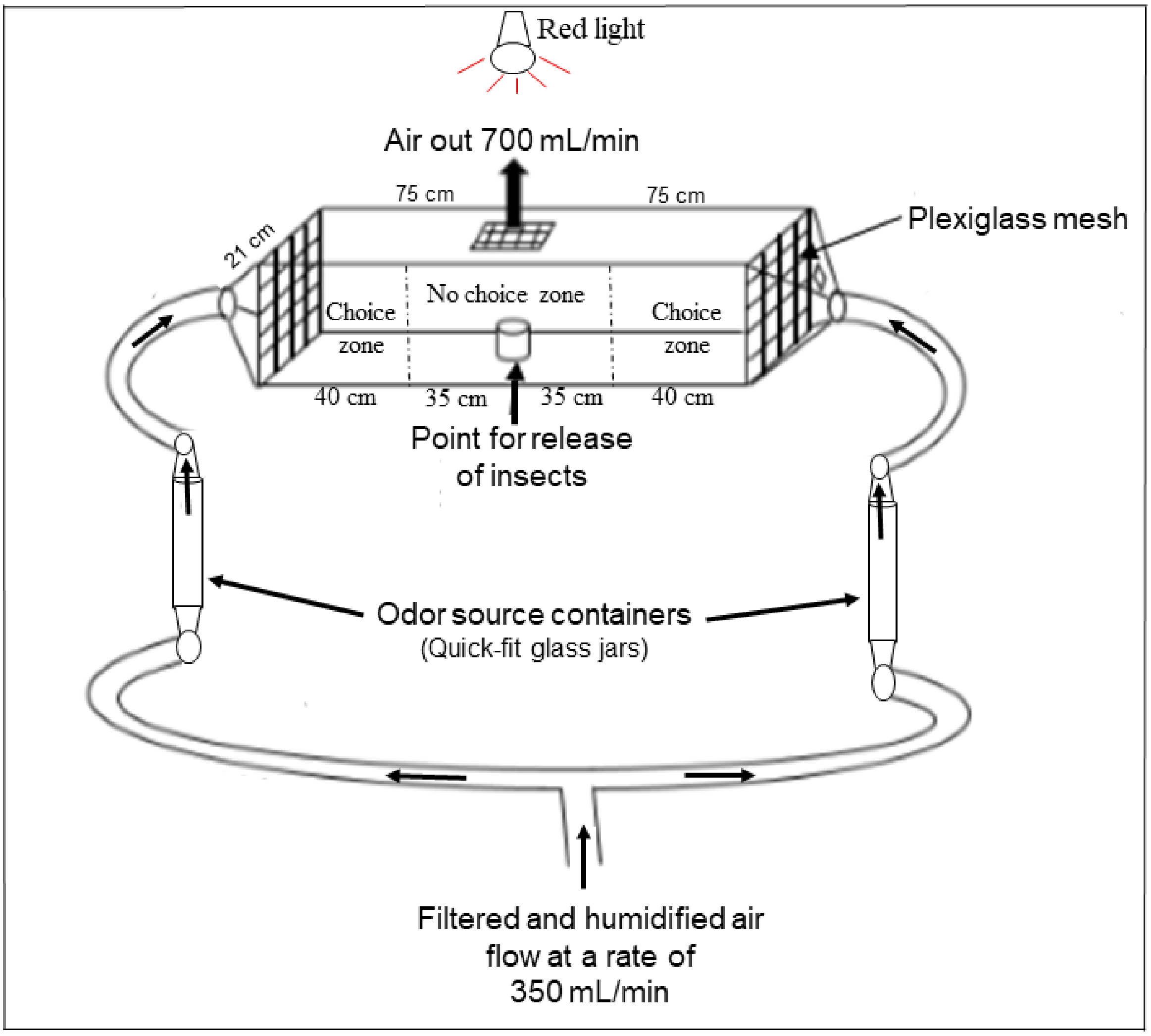

Figure S6 Design of the 2–choice wind tunnel used in the behavioral assays. For choice tests involving the use of plant, one of the two quick-fit jars (250 mL) was replaced by a cuboidal plexiglass cage (61 cm x 35 cm x 35 cm) that served as container for the plant.
